# Supplementary material for: Electron-donable heterojunctions with synergetic Ru-Cu pair sites for biocatalytic microenvironment modulations in inflammatory mandible defects
Source: Nat Commun. 2024 Nov 6;15:9592. doi: 10.1038/s41467-024-53824-y (PMC11541594; doi:10.1038/s41467-024-53824-y)
Supplement: Supplementary file 1 — Supplementary Information [file 41467_2024_53824_MOESM1_ESM.pdf]

Supplementary Information for

Electron-donable heterojunctions with synergetic Ru-Cu pair sites for biocatalytic microenvironment modulations in inflammatory mandible defects

This PDF file includes:

Supplementary Figures 1 to 53

Supplementary Tables 1 to 3

Other supporting materials for this manuscript include the following:

Supplementary Data 1 to 4

Supplementary Figures

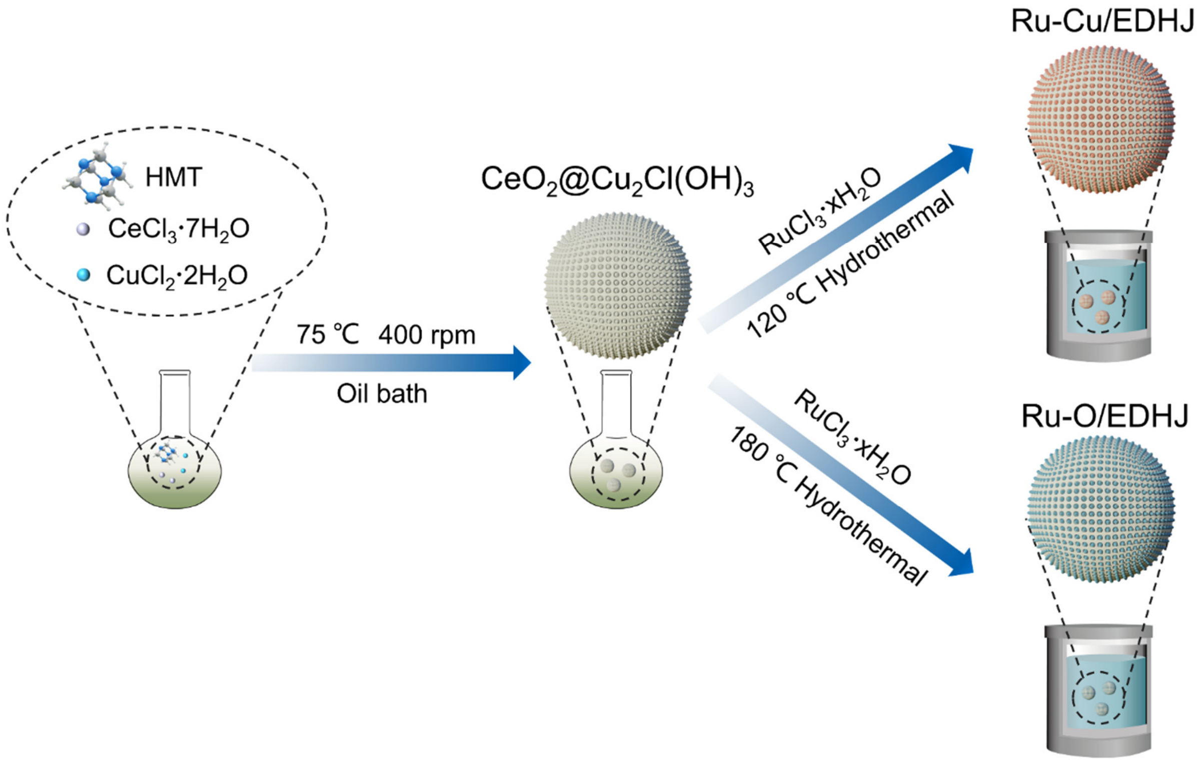

Supplementary Fig. 1. Schematic illustration of the synthetic process of $CeO_2@Cu_2Cl(OH)_3$ precursor, Ru-Cu/EDHJ, and Ru-O/EDHJ biocatalysts. HMT indicates the hexamethylenetetramine. Atomic color coding in the structure of HMT: N, blue; C, gray; H, white.

In our experiment, we started from the $CeO_2@Cu_2Cl(OH)_3$ precursor with abundant exposed surface OH species, which were synthesized by a two-step hydrothermal process as illustrated in Supplementary Fig. 1. We first uniformly dispersed $Cu_2Cl(OH)_3$ nanoclusters on the CeO_2 support. These $Cu_2Cl(OH)_3$ nanoclusters with surface -OH acts as functional nanoglues to localize metal atoms. Subsequent as-prepared $CeO_2@Cu_2Cl(OH)_3$ is added into a $RuCl_3$ solution, and strong electrostatic adsorption deposits Ru atoms on the $Cu_2Cl(OH)_3$. By judiciously changing the reaction temperature (120 °C and 180 °C), the atomically dispersed Ru atoms are deposited on two supports, $CeO_2@Cu_2O$ (EDHJ) and $CeO_2@CuO$, which are named Ru-Cu/EDHJ and Ru-O/EDHJ, respectively.

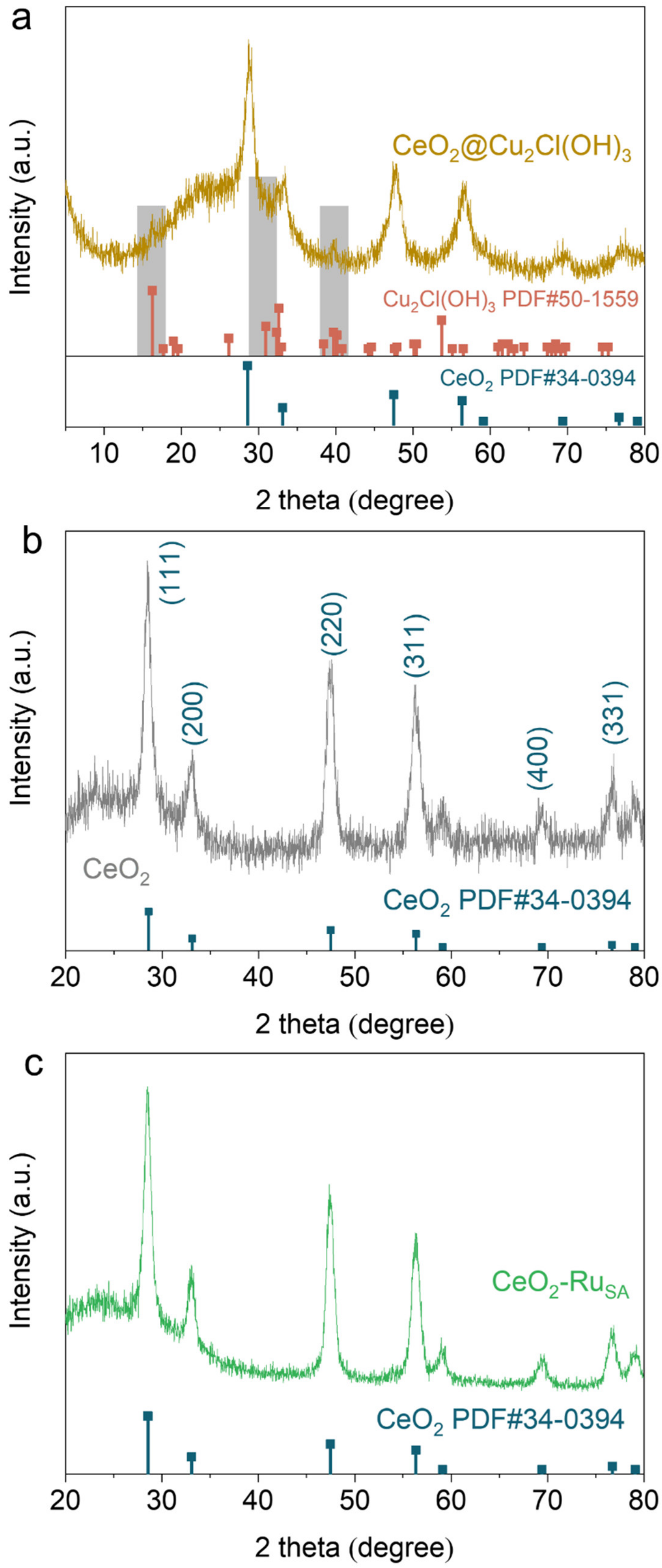

Supplementary Fig. 2. X-ray diffraction (XRD) patterns of **a** $CeO_2@Cu_2Cl(OH)_3$ precursor, **b** CeO_2, and **c** CeO_2-Ru_{SA}. Source data are provided as a Source Data file.

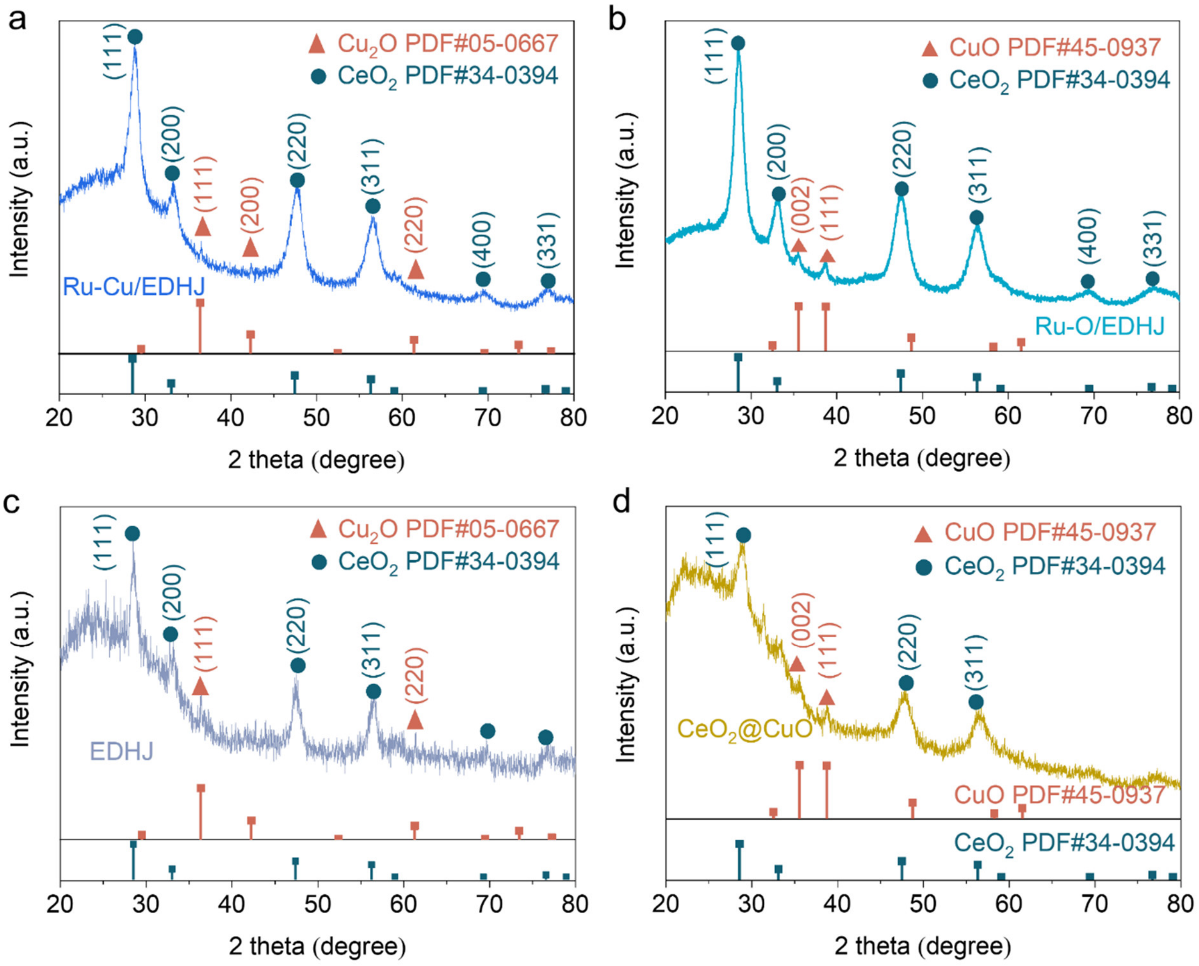

Supplementary Fig. 3. XRD patterns of **a** Ru-Cu/EDHJ, **b** Ru-O/EDHJ, **c** EDHJ, and **d** CeO_2@CuO. Source data are provided as a Source Data file.

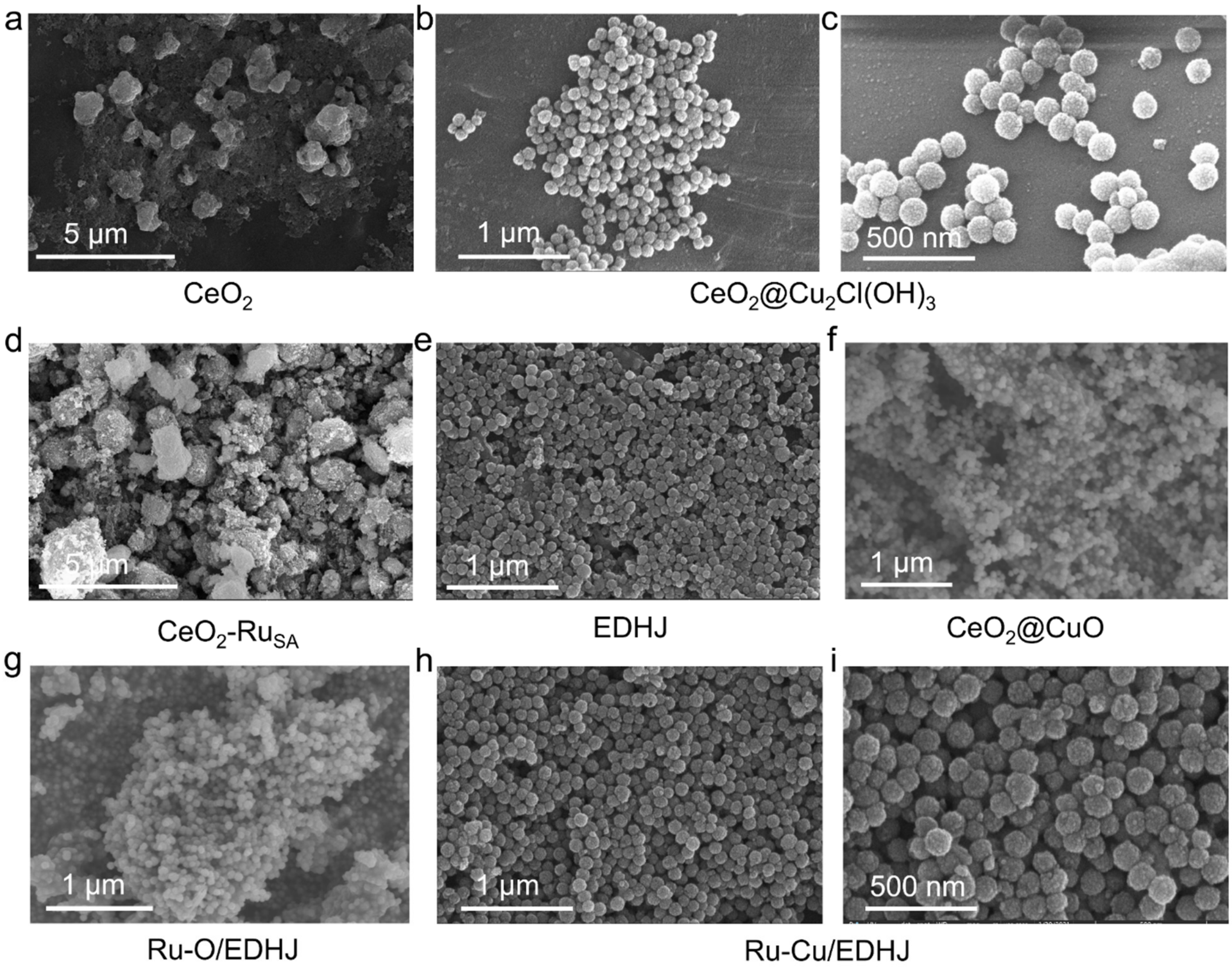

Supplementary Fig. 4. Scanning electron microscope (SEM) images of **a** CeO_2, **b-c** $CeO_2@Cu_2Cl(OH)_3$, **d** CeO_2-Ru_{SA}, **e** EDHJ, **f** $CeO_2@CuO$, **g** Ru-O/EDHJ, and **h-i** Ru-Cu/EDHJ.

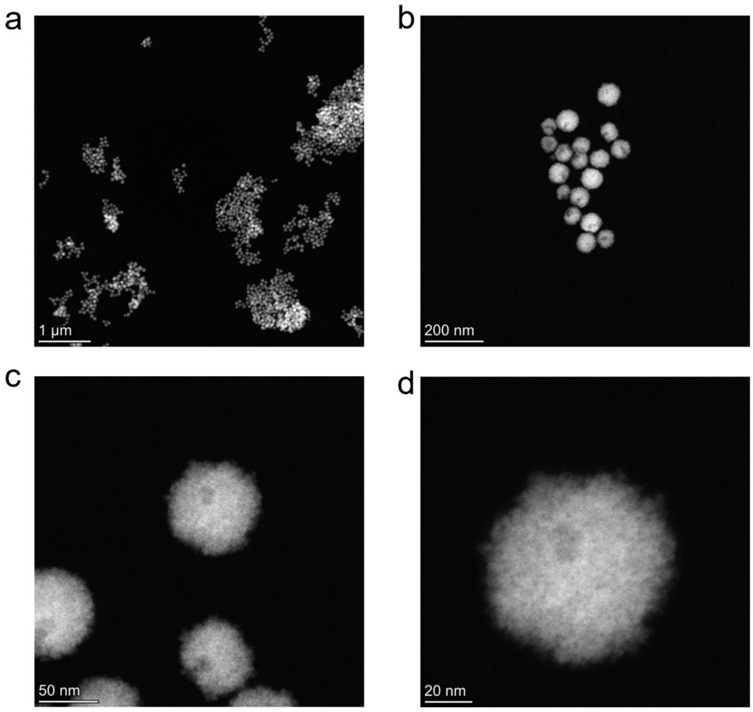

Supplementary Fig. 5. High-angle annular dark-field scanning transmission electron microscopy (HAADF-STEM) images of Ru-Cu/EDHJ at varying magnifications: **a** 1 μm, **b** 200 nm, **c** 50 nm, and **d** 20 nm.

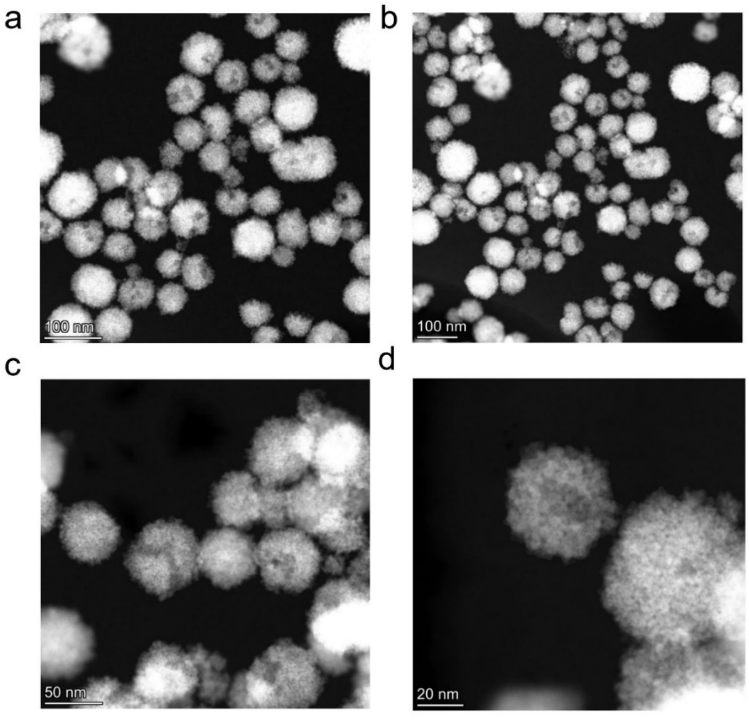

Supplementary Fig. 6. HAADF-STEM images of Ru-O/EDHJ at varying magnifications: **a** 100 nm, **b** 100 nm, **c** 50 nm, and **d** 20 nm.

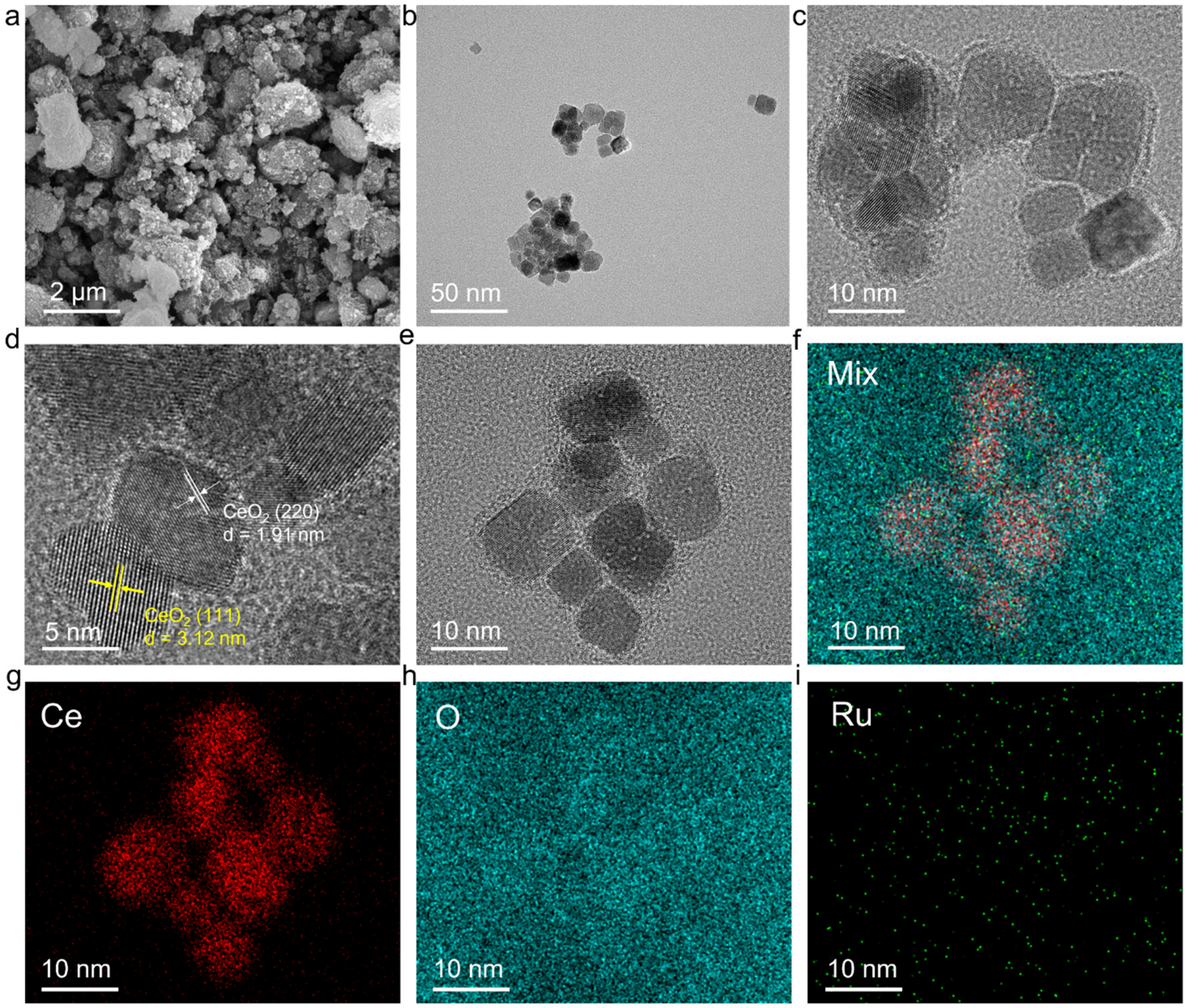

Supplementary Fig. 7. a SEM image, **b-e** high-resolution TEM images at varying magnifications, and **f-i** corresponding energy-dispersive X-ray spectroscopy (EDS) mapping of CeO_2-Ru_{SA}.

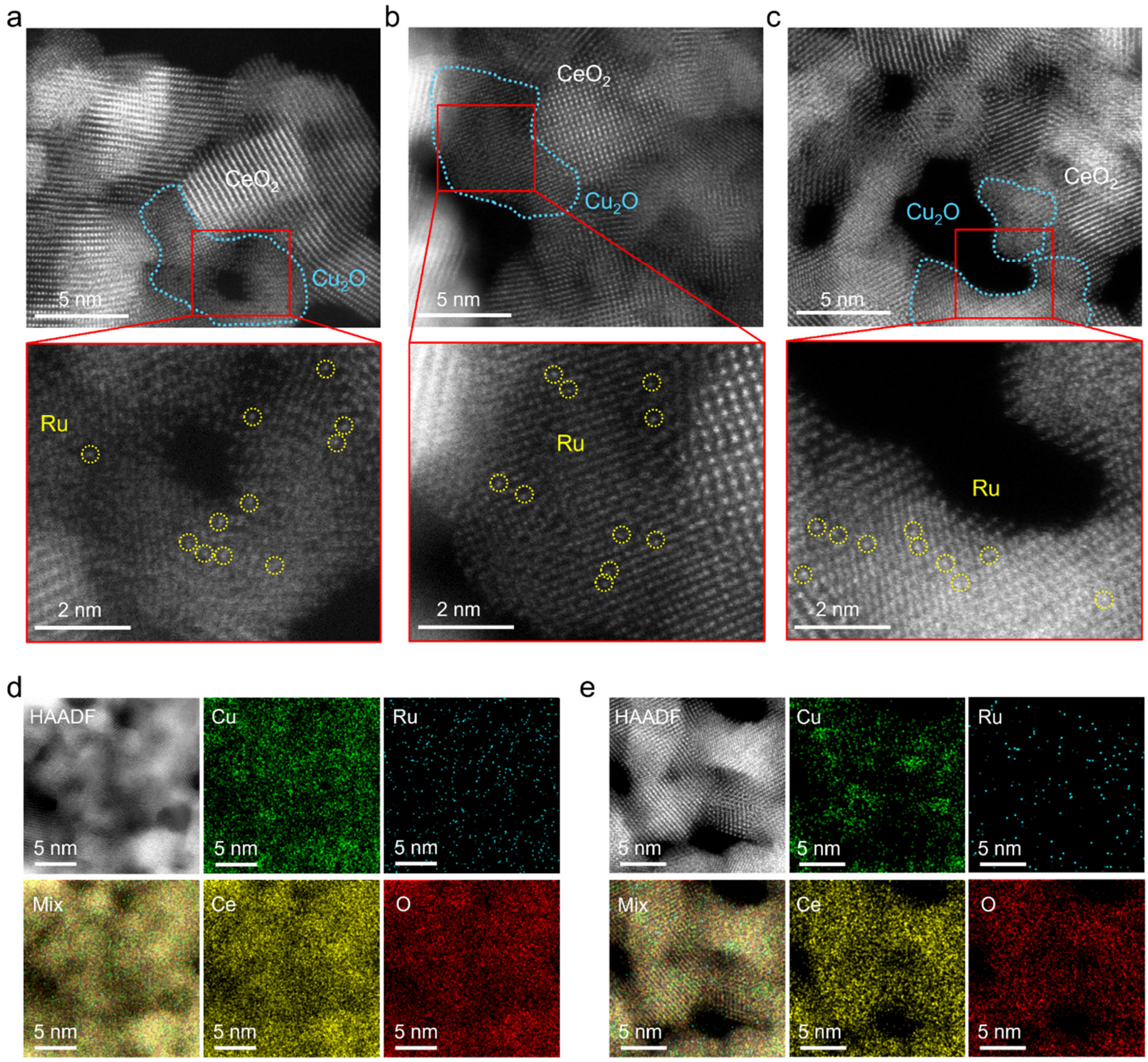

Supplementary Fig. 8. a-c Atomic-scale HAADF-STEM images and **d-e** EDS mapping of Ru-Cu/EDHJ.

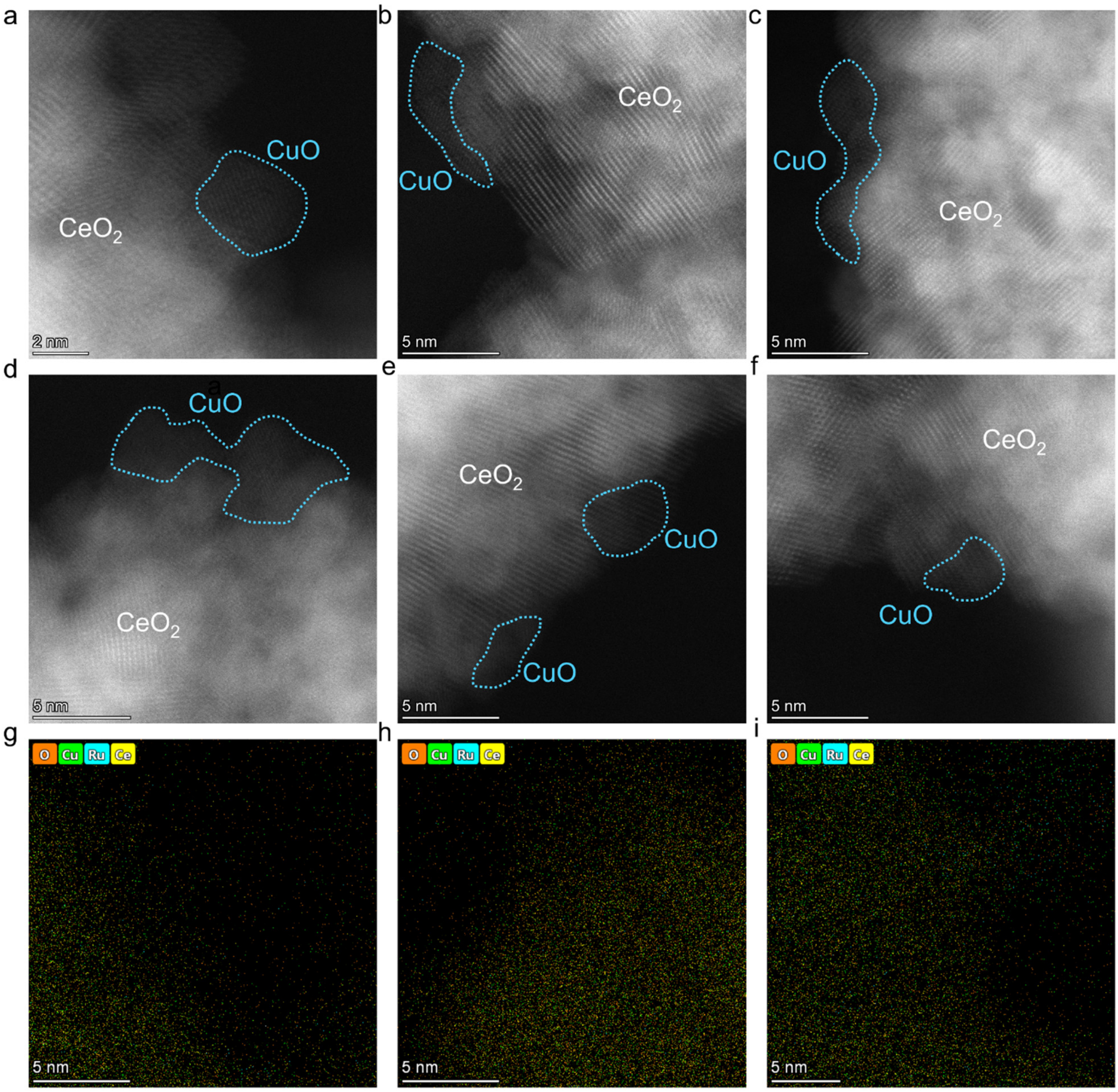

Supplementary Fig. 9. a-f Atomic-scale HAADF-STEM images and **g-i** corresponding EDS mapping of Ru-O/EDHJ.

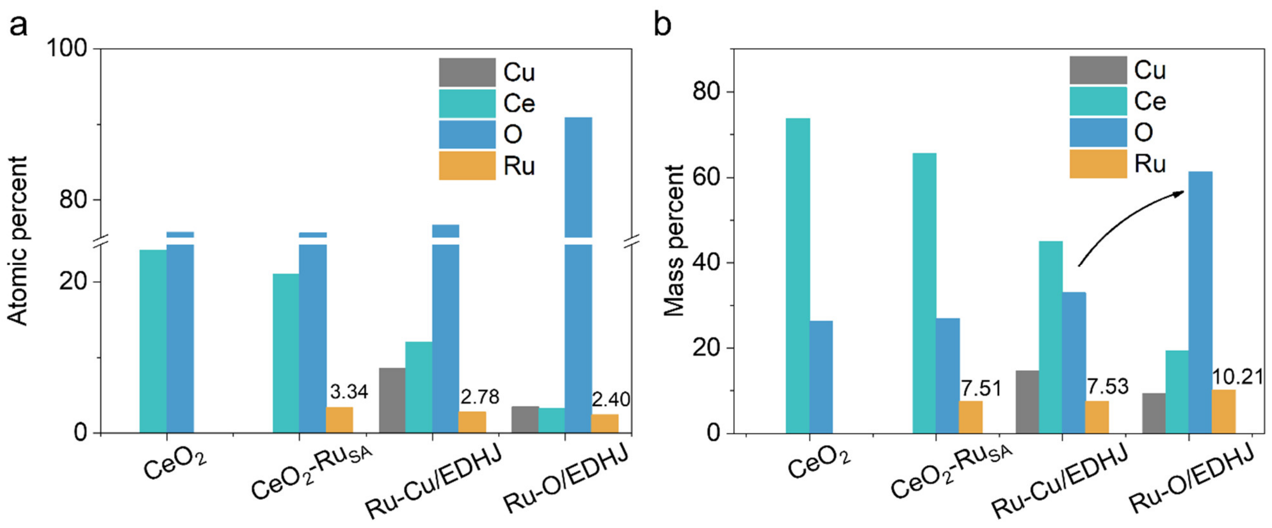

Supplementary Fig. 10. Elemental compositions of CeO_2, CeO_2-Ru_{SA}, Ru-Cu/EDHJ, and Ru-O/EDHJ on X-ray photoelectron spectroscopy (XPS) data, **a** atomic percent and **b** mass percent. Source data are provided as a Source Data file.

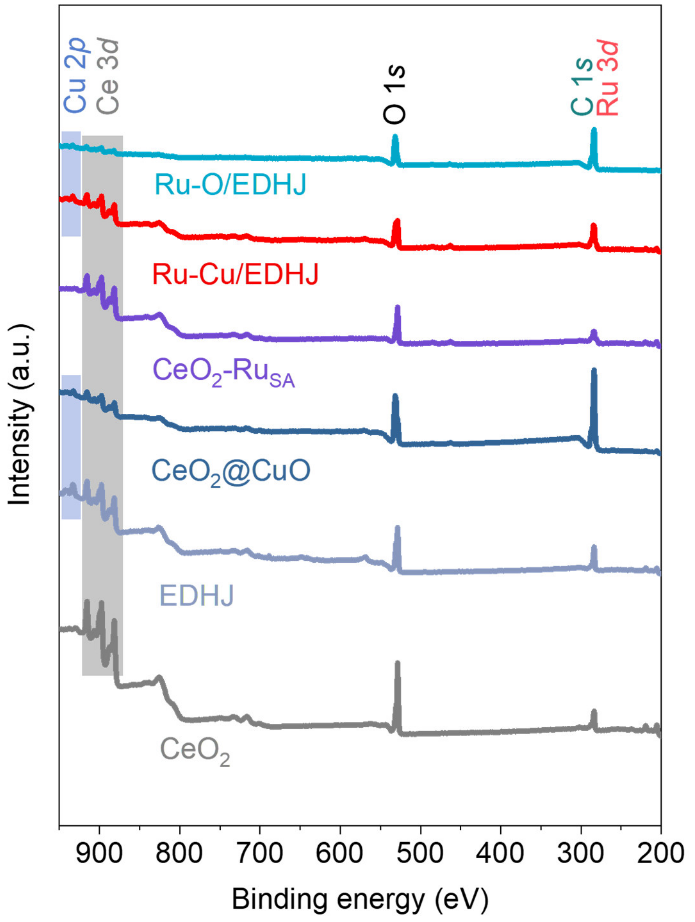

Supplementary Fig. 11. XPS survey spectra of CeO_2, EDHJ, CeO_2@CuO, CeO_2-Ru_{SA}, Ru-Cu/EDHJ, and Ru-O/EDHJ. Source data are provided as a Source Data file.

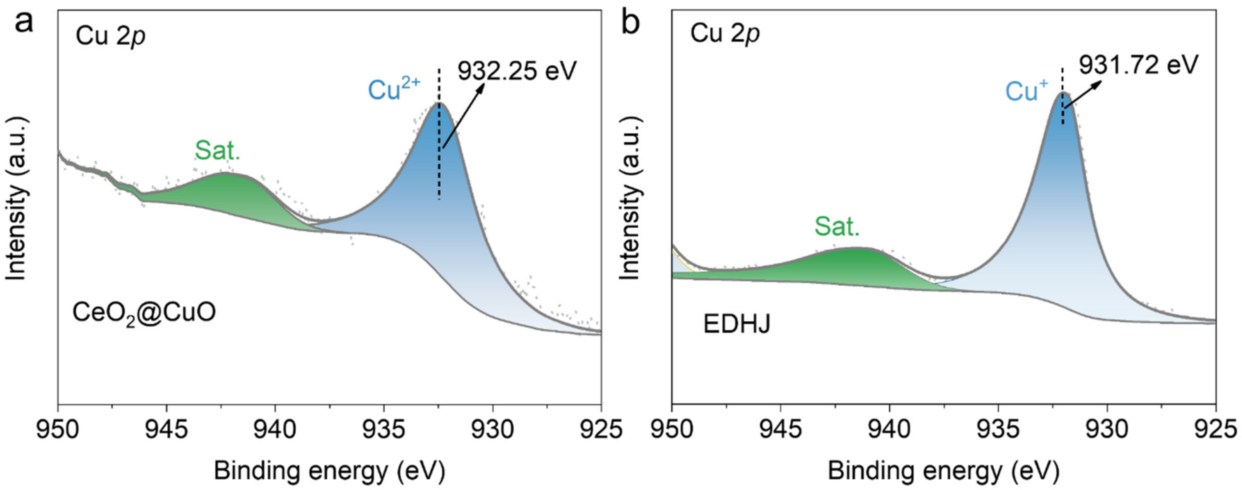

Supplementary Fig. 12. Cu 2*p* XPS spectra of **a** CeO_2@CuO and **b** EDHJ. Sat. indicates the satellite peaks. Source data are provided as a Source Data file.

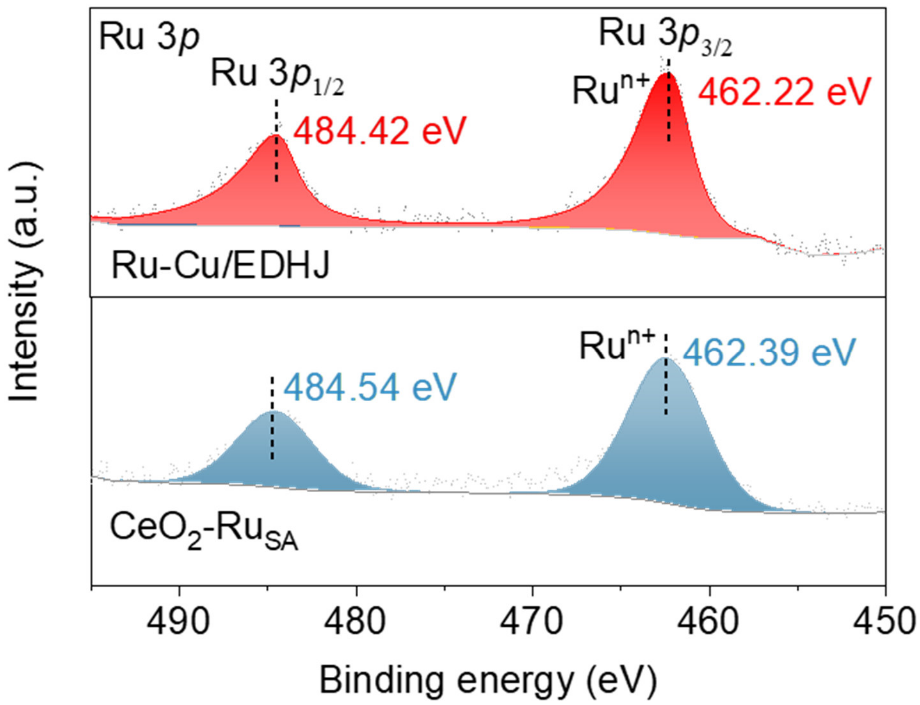

Supplementary Fig. 13. Ru 3*p* XPS spectra of CeO_2-Ru_{SA} and Ru-Cu/EDHJ. Source data are provided as a Source Data file.

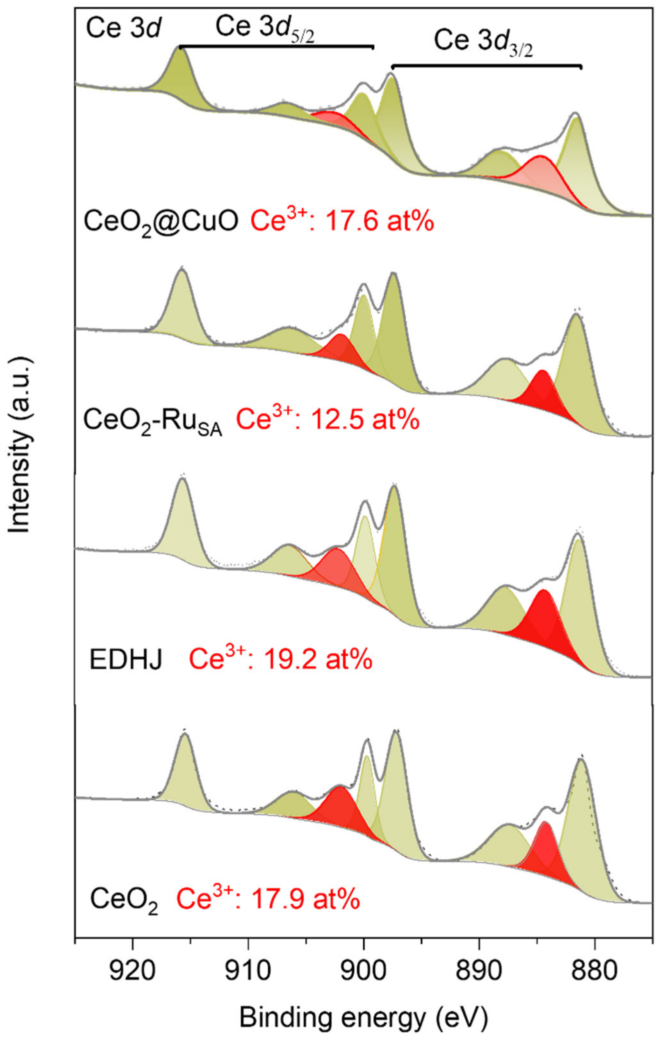

Supplementary Fig. 14. Ce 3*d* XPS spectra of CeO_2, EDHJ, CeO_2-Ru_{SA}, and CeO_2@CuO. Source data are provided as a Source Data file.

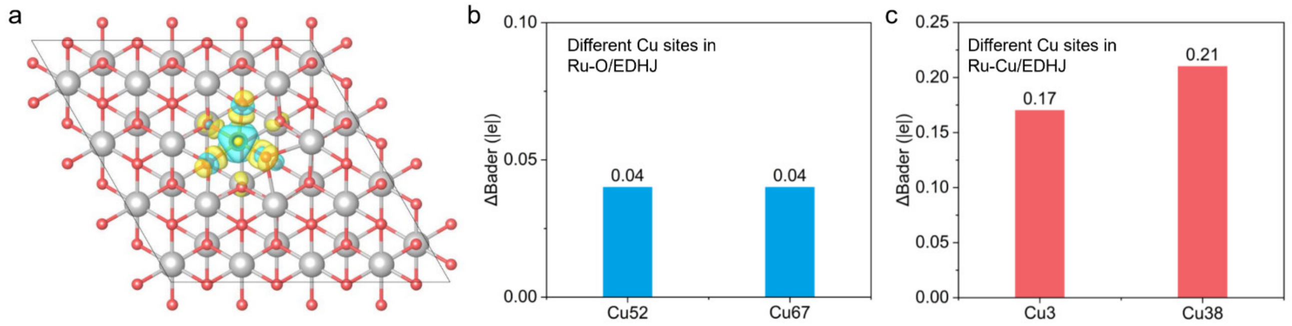

Supplementary Fig. 15. a Differential charge density analysis of Ru in CeO_2-Ru_{SA} (cyan and yellow represent charge depletion and accumulation, respectively, the cut-off of the density-difference isosurface is 0.006 e·$Bohr^{-3}$). Atomic color coding in the structure: Ru, yellow; Ce, white; O, red. The

relative change values in Bader charge of Cu sites in **b** Ru-O/EDHJ and **c** Ru-Cu/EDHJ before and after Ru doping. Source data are provided as a Source Data file.

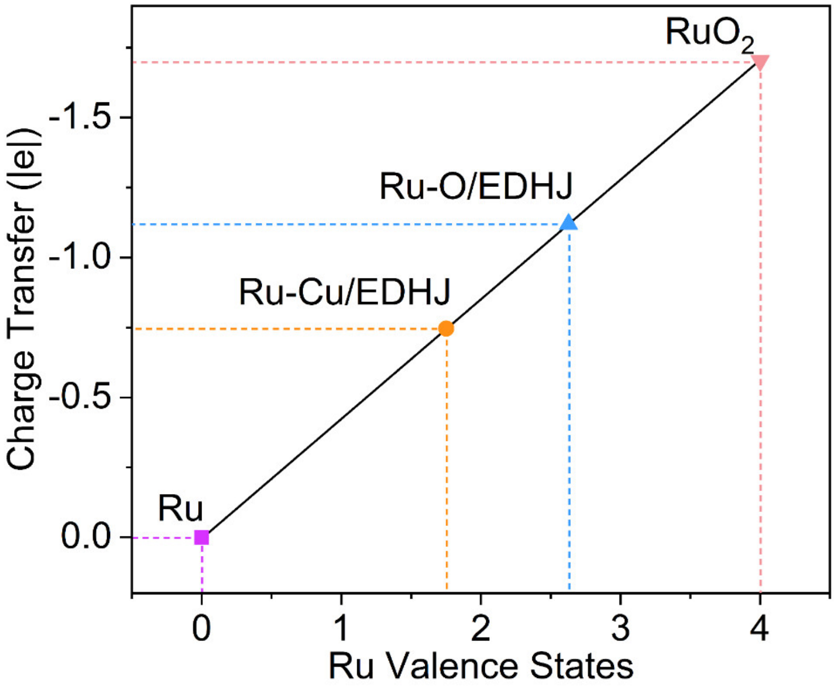

Supplementary Fig. 16. Ru valence states analysis of different samples. Source data are provided as a Source Data file.

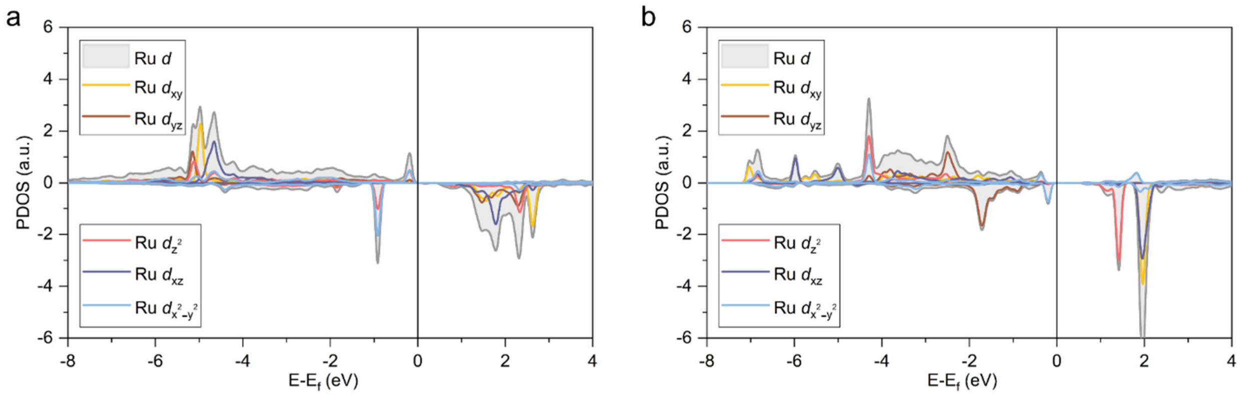

Supplementary Fig. 17. Partial density of states (PDOS) analysis of Ru 4*d* orbital of **a** Ru-O/EDHJ and **b** Ru-Cu/EDHJ. Source data are provided as a Source Data file.

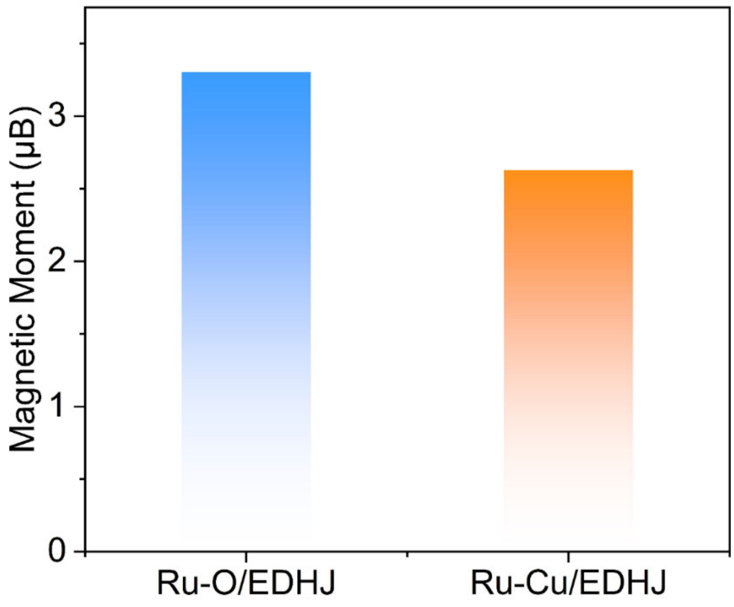

Supplementary Fig. 18. Calculated magnetic moment statistics of Ru of Ru-O/EDHJ and Ru-Cu/EDHJ. Source data are provided as a Source Data file.

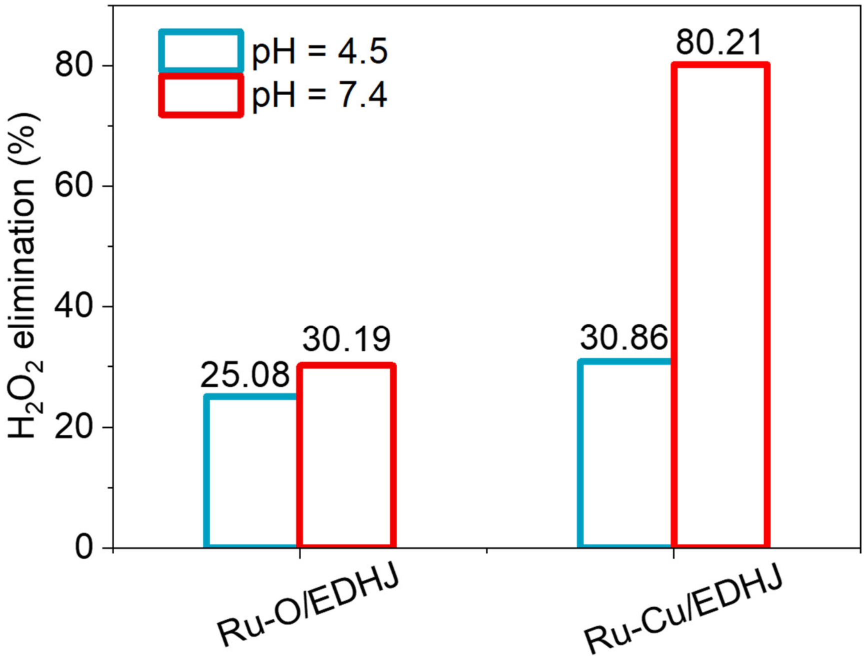

Supplementary Fig. 19. H_2O_2 elimination ratio of Ru-O/EDHJ and Ru-Cu/EDHJ at different pH values. Experiments were repeated independently three times with similar results. Source data are provided as a Source Data file.

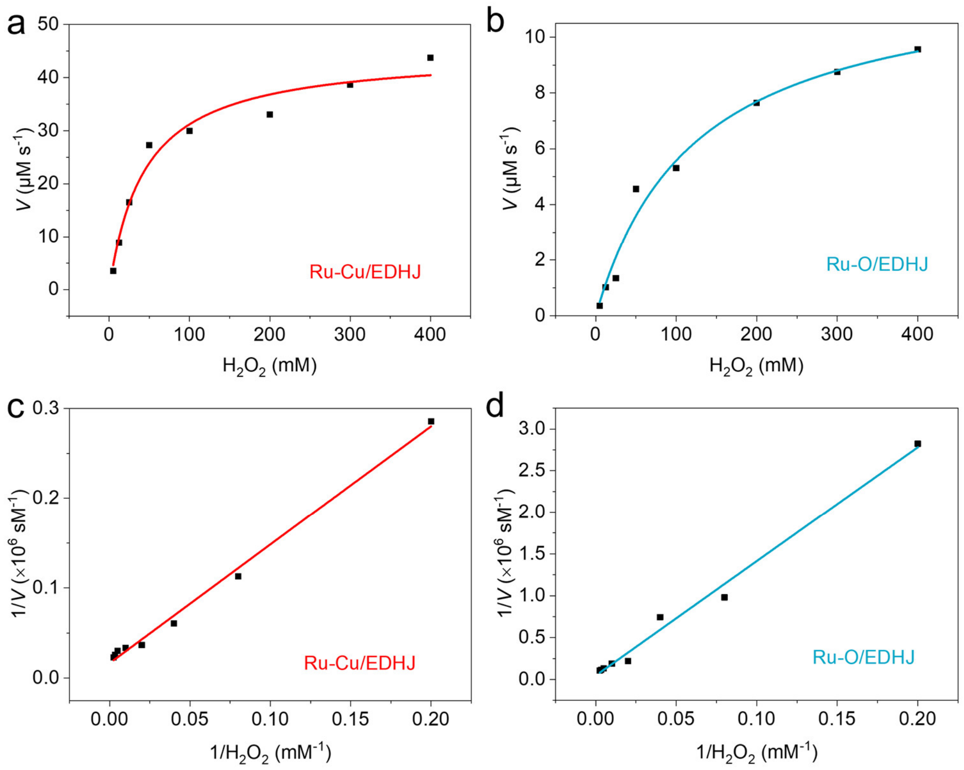

Supplementary Fig. 20. a-b Michaelis–Menten kinetic analysis and **c-d** Lineweaver–Burk plot for Ru-Cu/EDHJ, and Ru-O/EDHJ with H_2O_2 as substrate. Source data are provided as a Source Data file.

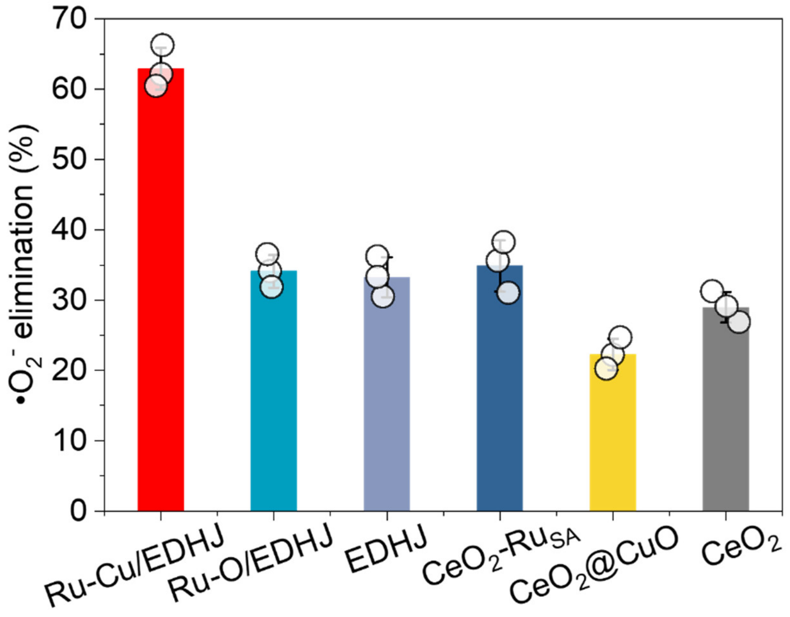

Supplementary Fig. 21. Superoxide dismutase (SOD)-like activity of different catalysts. Experiments were repeated independently three times with similar results. n = 3 independent replicates. All error bars represent mean ± SD. Source data are provided as a Source Data file.

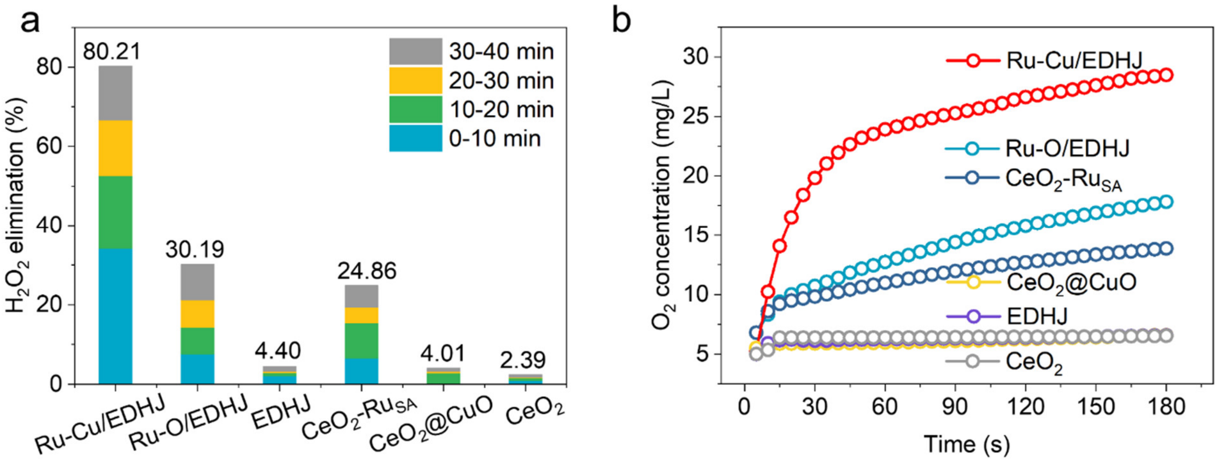

Supplementary Fig. 22. **a** H_2O_2 elimination ratio of different catalysts. **b** The O_2 concentration produced by different catalysts was measured by an oxygen dissolving meter in the presence of H_2O_2. Experiments were repeated independently three times with similar results. Source data are provided as a Source Data file.

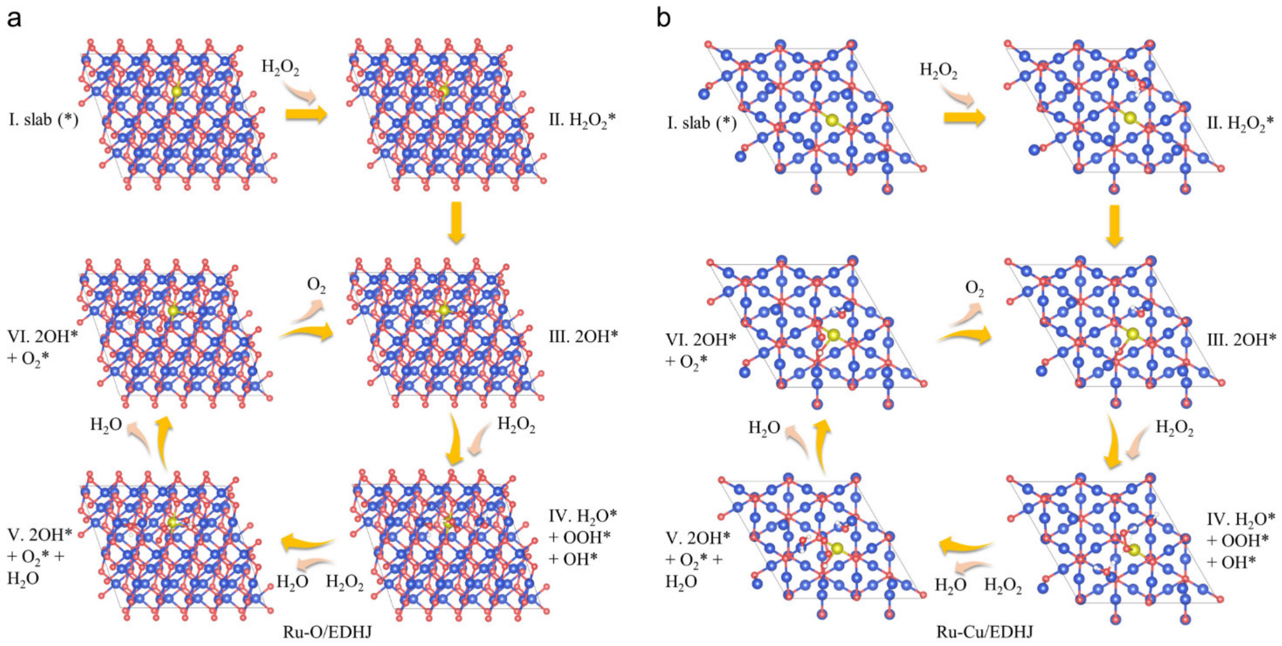

Supplementary Fig. 23. Proposed catalase (CAT)-like catalytic reaction pathways of **a** Ru-O/EDHJ and **b** Ru-Cu/EDHJ. Atomic color coding in the structure: Ru, yellow; Cu, blue; O, red; and H, white.

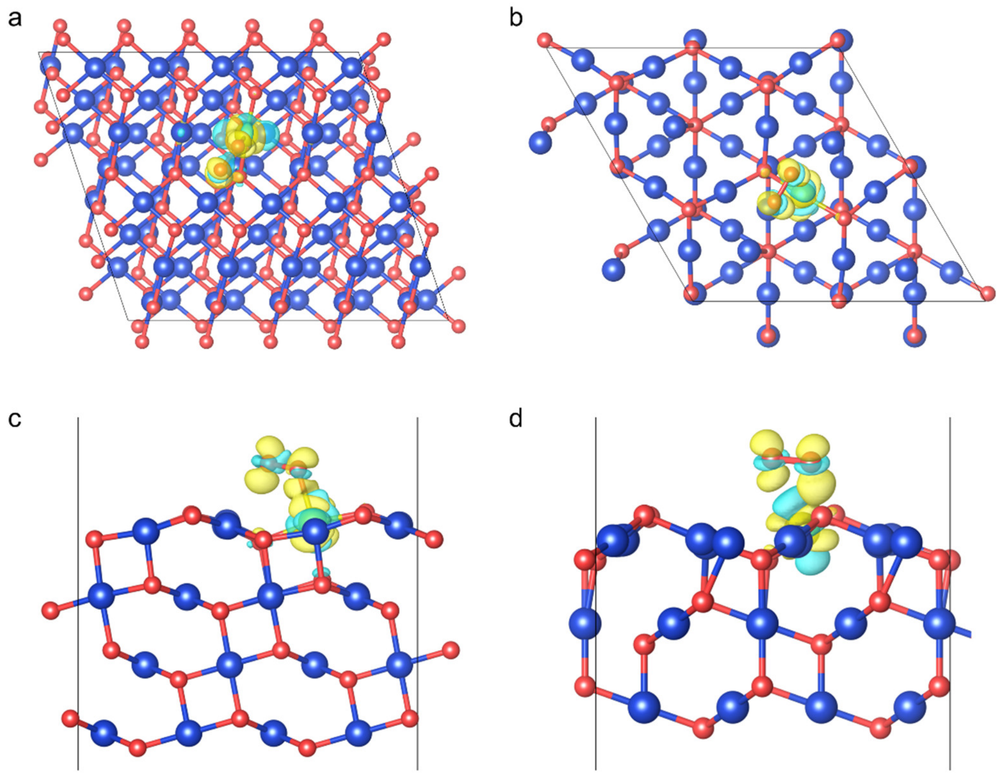

Supplementary Fig. 24. Differential charge density analysis of O_2* in **a, c** Ru-O/EDHJ and **b, d** Ru-Cu/EDHJ (cyan and yellow represent charge depletion and accumulation, respectively, the cut-off of the density-difference isosurface is 0.006 e·$Bohr^{-3}$). Atomic color coding in the structure: Ru, yellow; Cu, blue; and O, red.

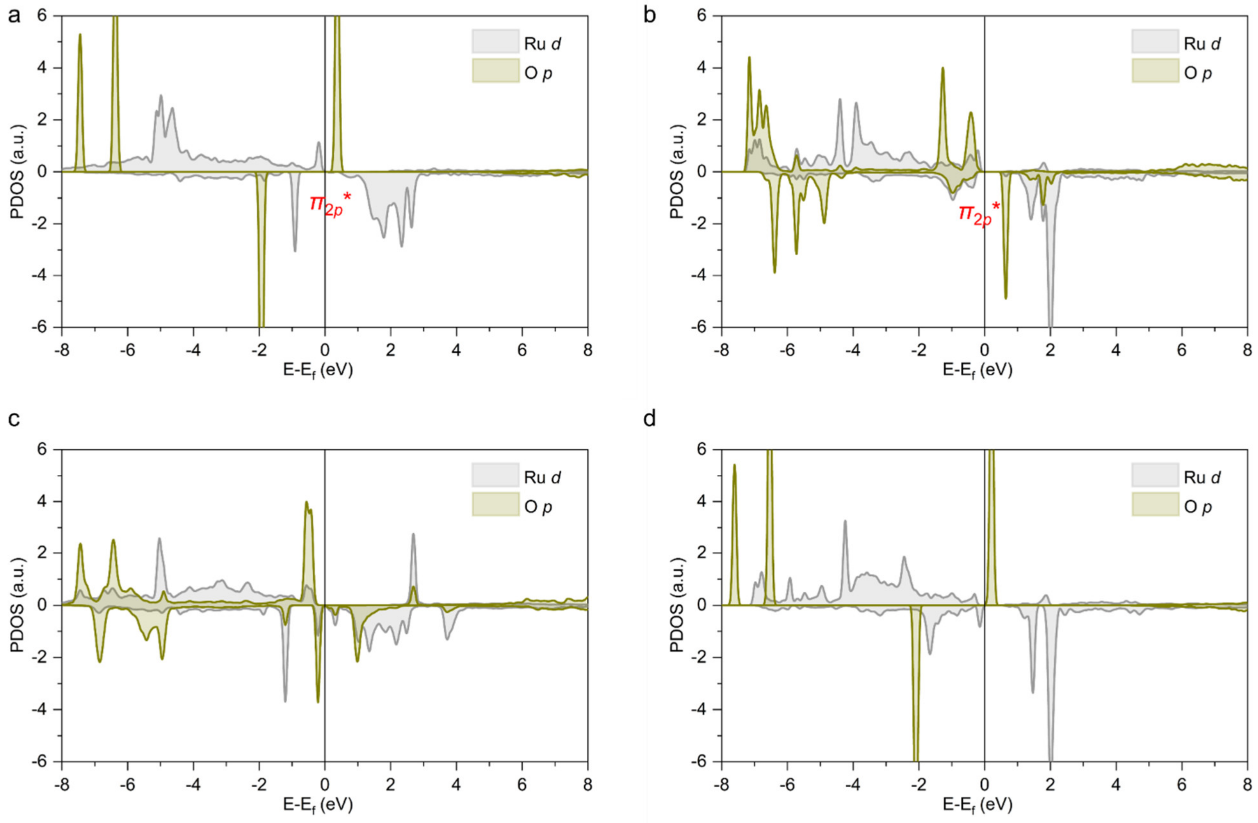

Supplementary Fig. 25. Calculated PDOS **a-b** before (M-O_2) and **c-d** after (M*O_2) the interaction of O_2 with **a, c** Ru-O/EDHJ and **b, d** Ru-Cu/EDHJ. Source data are provided as a Source Data file.

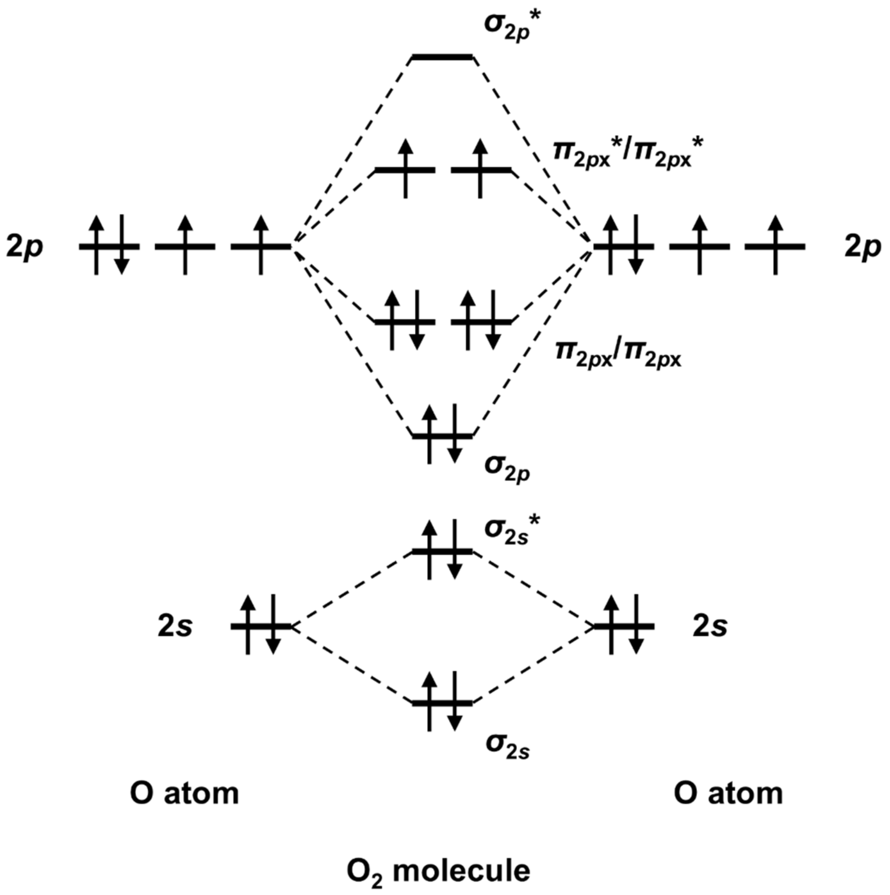

Supplementary Fig. 26. Molecular orbital diagram of O_2.

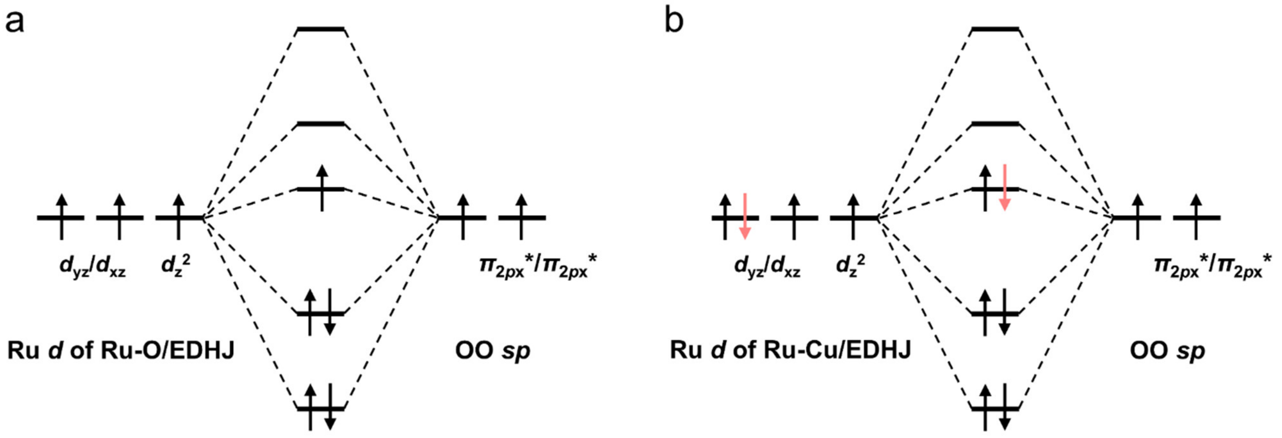

Supplementary Fig. 27. Molecular orbital diagrams of Ru-O_2* of **a** Ru-O/EDHJ and **b** Ru-Cu/EDHJ.

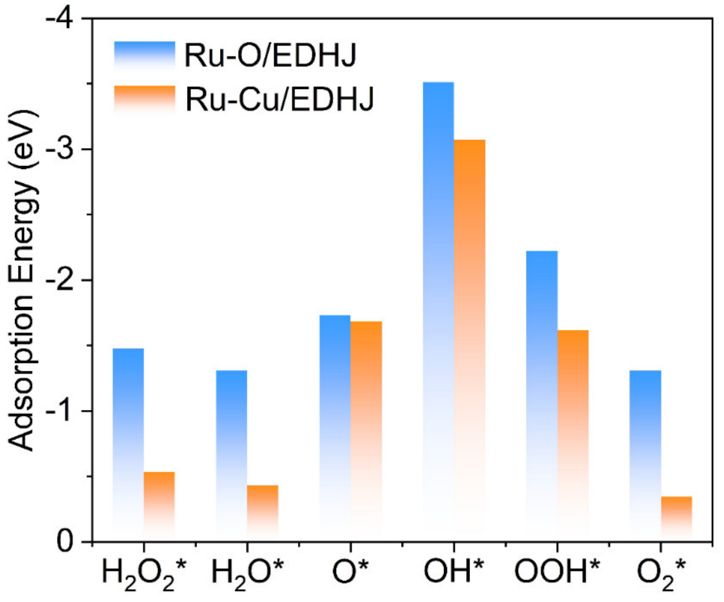

Supplementary Fig. 28. Calculated adsorption energy of Ru-O/EDHJ and Ru-Cu/EDHJ with different oxygen-containing intermediates. Source data are provided as a Source Data file.

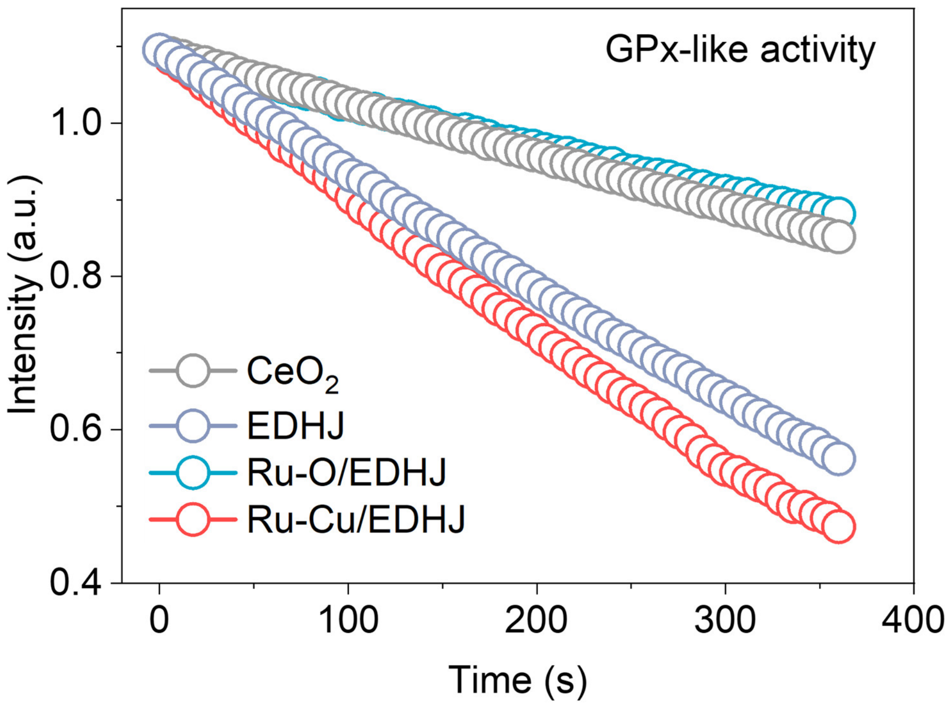

Supplementary Fig. 29. Time-dependent glutathione peroxidase (GPx)-like activity of different materials. Experiments were repeated independently three times with similar results. Source data are provided as a Source Data file.

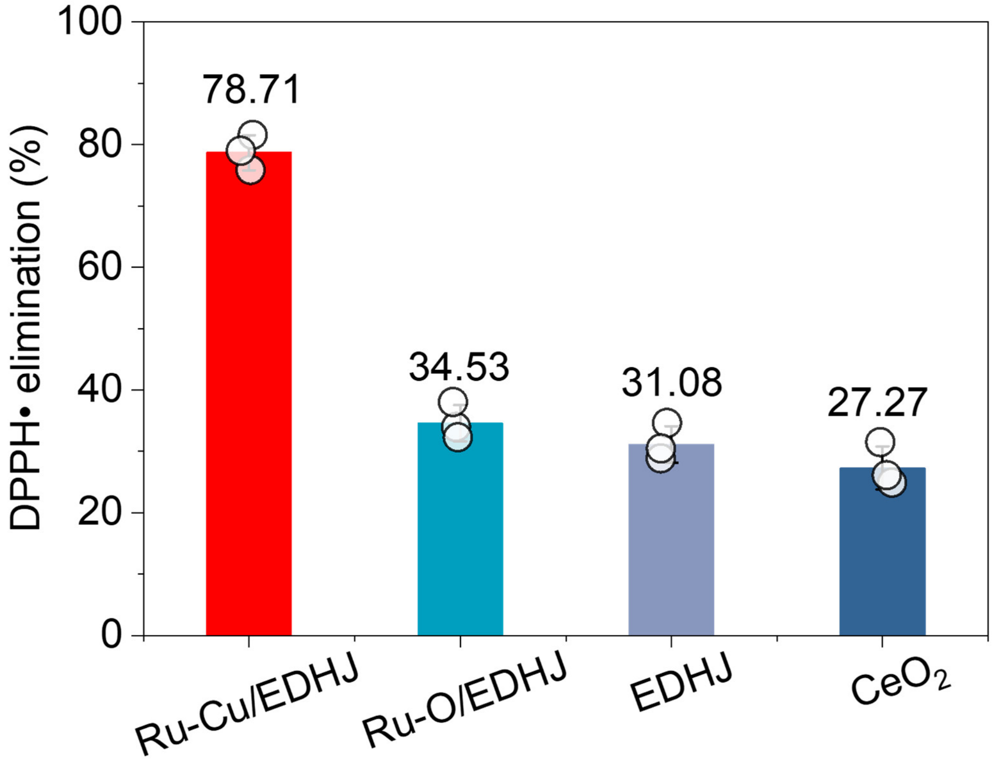

Supplementary Fig. 30. 1,1-diphenyl-2-picryl-hydrazyl radical (DPPH•) elimination rate of Ru-Cu/EDHJ, Ru-O/EDHJ, EDHJ, and CeO_2. Experiments were repeated independently three times with similar results. n = 3 independent replicates. All error bars represent mean ± SD. Source data are provided as a Source Data file.

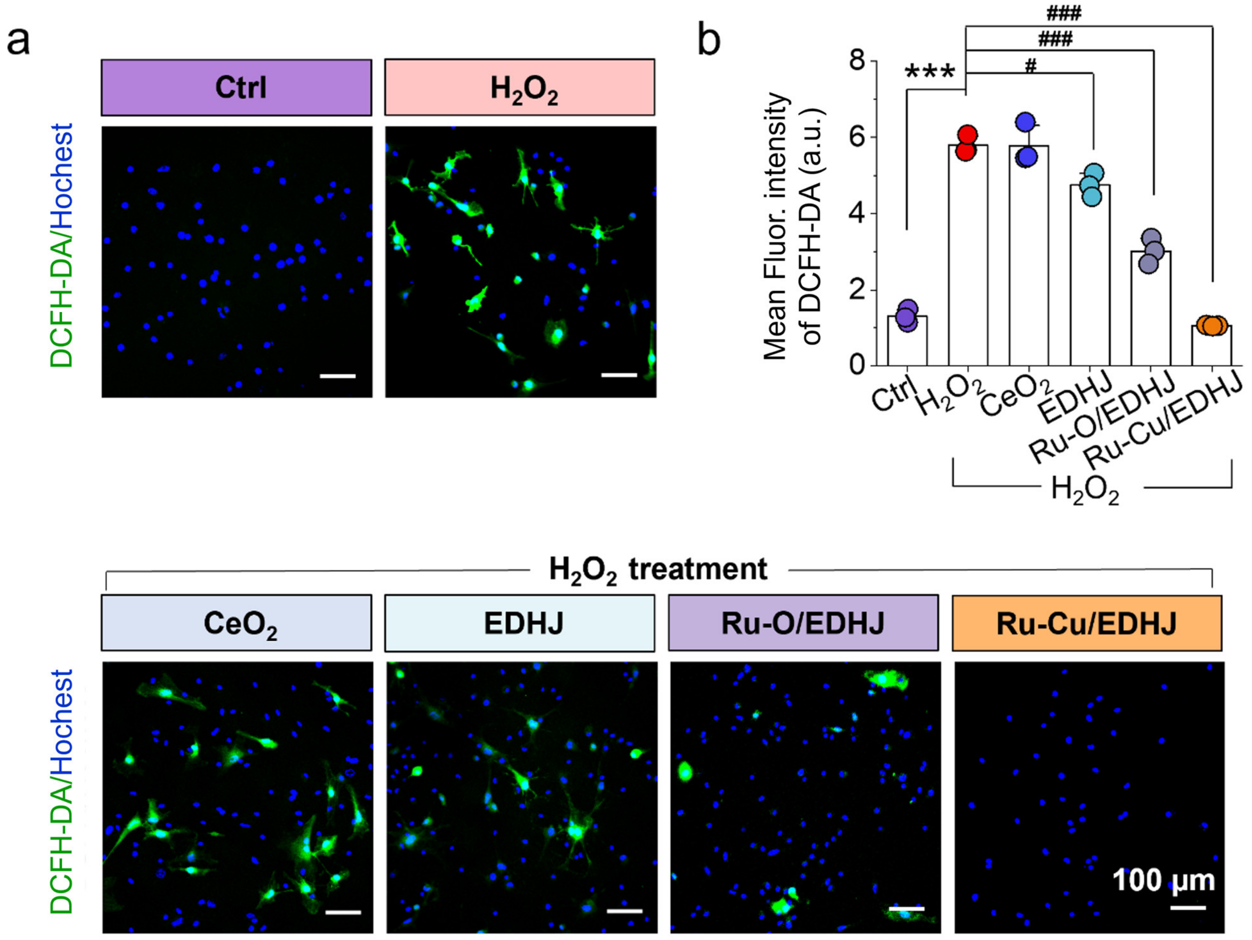

Supplementary Fig. 31. **a** Fluorescence images, Ctrl (PBS), **b** and mean fluorescence intensity of 2,7-dichlorodihydrofluorescein diacetate (DCFH-DA) staining of ROS in mMSCs (n = 3 independent replicates), $p_{(H2O2)} < 0.0001$, $p_{(EDHJ\text{-}H2O2)} = 0.0144$, $p_{(Ru\text{-}O/EDHJ\text{-}H2O2)} < 0.0001$, $p_{(Ru\text{-}Cu/EDHJ\text{-}H2O2)} < 0.0001$. Scale bars: 100 µm. Data are presented as means ± SD., $^{***}p < 0.001$, $^{\#}p < 0.05$, $^{\#\#\#}p < 0.001$; one-way ANOVA with multiple comparisons test. Source data are provided as a Source Data file.

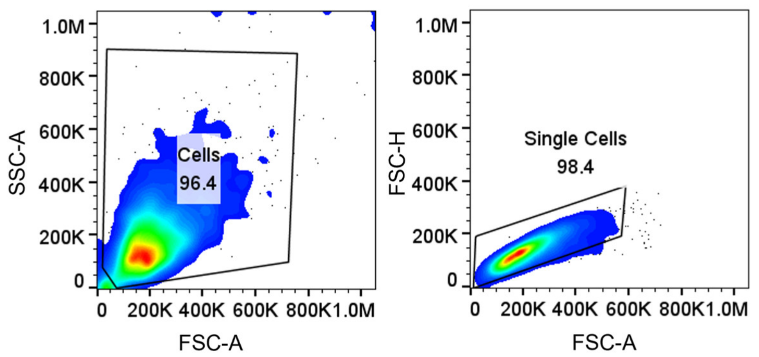

Supplementary Fig. 32. A Gating strategy for Fig. 5b.

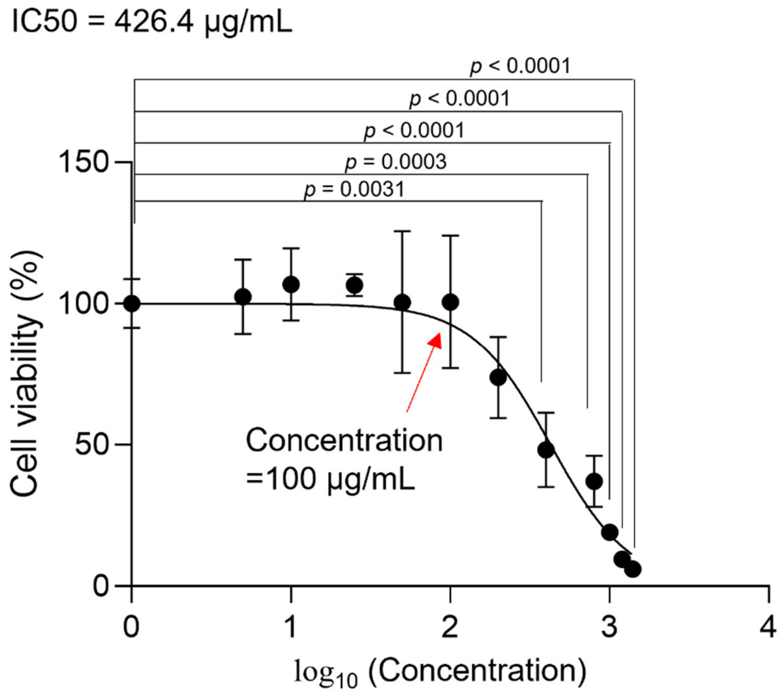

Supplementary Fig. 33. Quantitative analysis of cell viability for Ru-Cu/EDHJ without H_2O_2 using the Cell Counting Kit-8 (CCK-8), inhibitory concentration (IC50) = 426.4 µg/mL (n = 3 independent replicates). Data are presented as means ± SD., one-way ANOVA with multiple comparisons test. Source data are provided as a Source Data file.

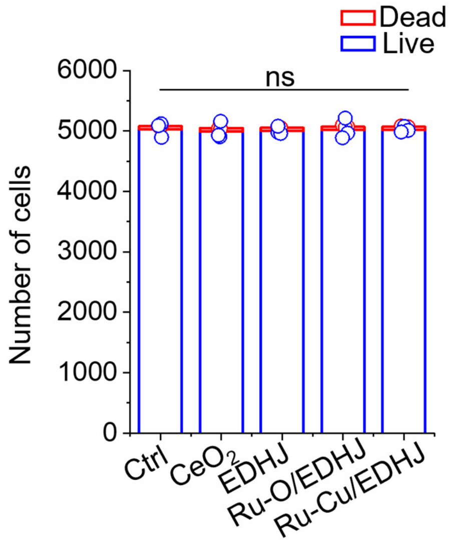

Supplementary Fig. 34. The number of cells with different materials counted after Live/Dead staining (n = 3 independent replicates). Data are presented as means ± SD.; ns indicates no significance; one-way ANOVA with multiple comparisons test. Source data are provided as a Source Data file.

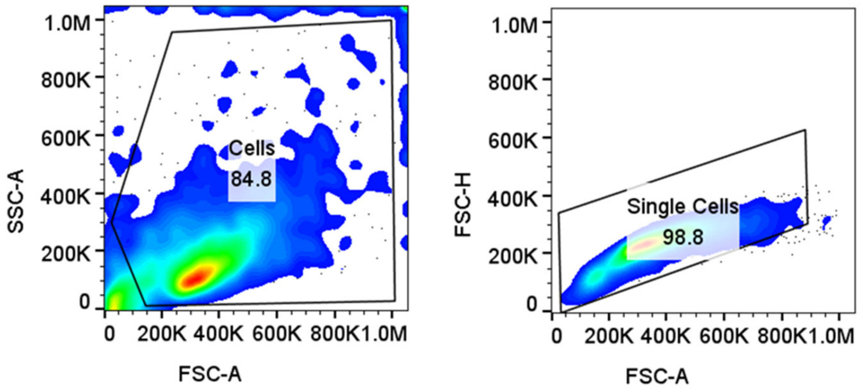

Supplementary Fig. 35. A Gating strategy for Fig. 6g.

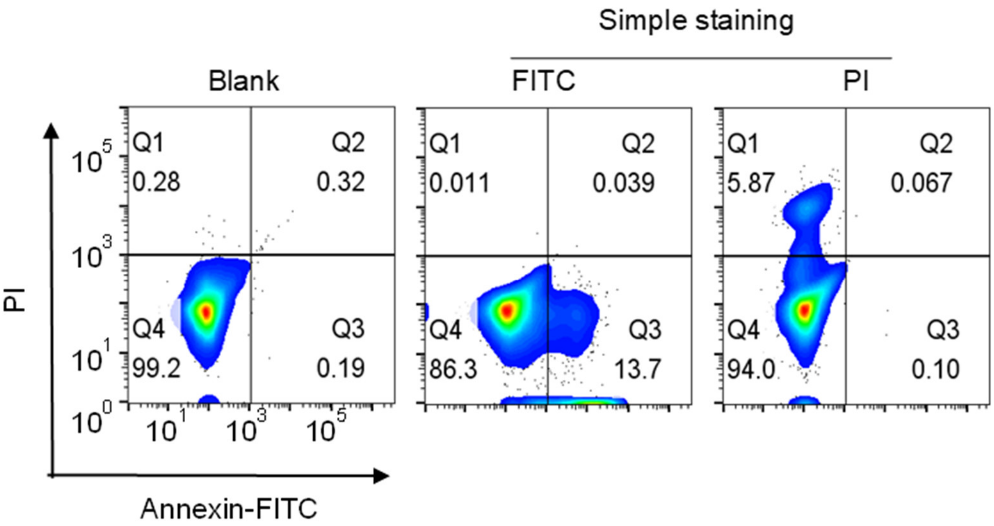

Supplementary Fig. 36. Apoptosis analysis by flow cytometry of Annexin V-FITC/PI stained hMSCs. The blank group indicates no staining; FITC and PI groups are single-dye staining with FITC and PI, respectively (n = 3 independent replicates).

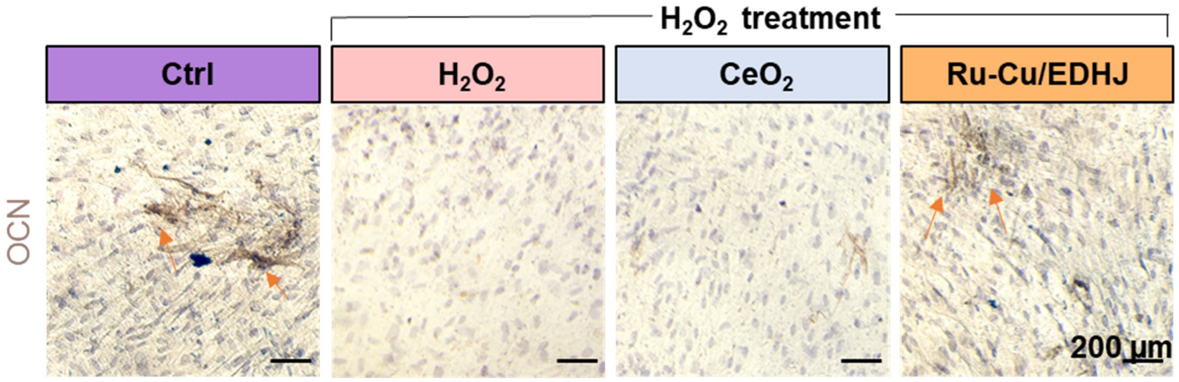

Supplementary Fig. 37. Immunohistochemical staining images of osteocalcin (OCN) in hMSCs, orange arrow indicates the positive expression area (n = 3 independent replicates). Scale bars: 200 μm.

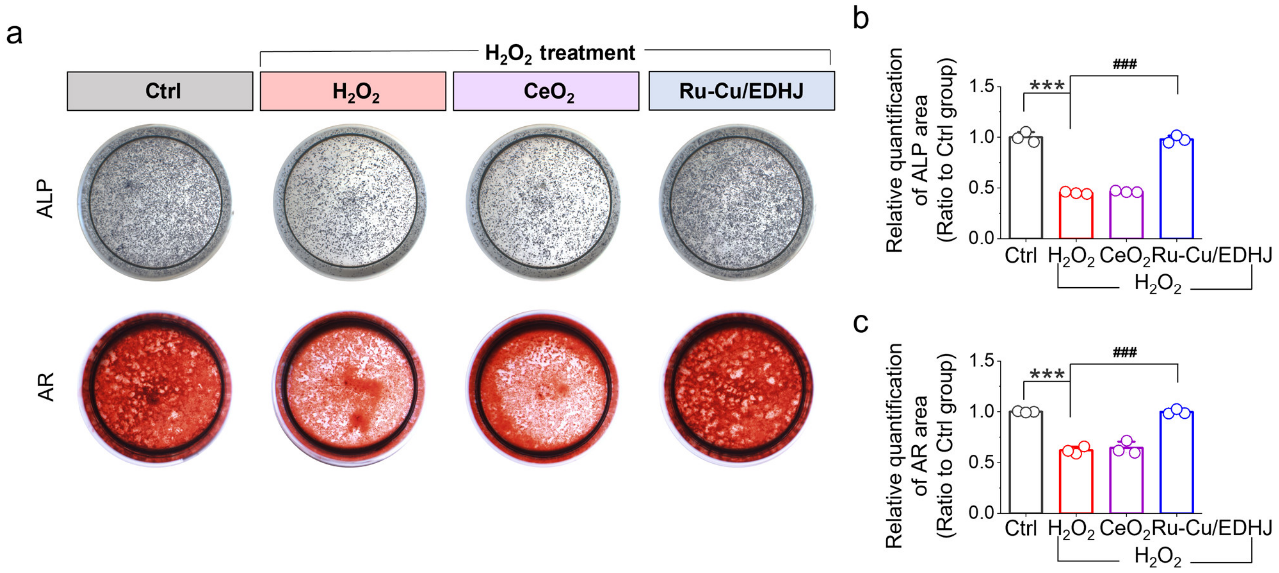

Supplementary Fig. 38. a Alkaline phosphatase (ALP) staining after 3-day *in vitro* osteo-induction and alizarin red (AR) staining after 21-day *in vitro* osteo-induction using the mMSCs. Quantitative analysis of **b** ALP (n = 3 independent replicates), $p_{(H2O2)} < 0.0001$, $p_{(Ru\text{-}Cu/EDHJ\text{-}H2O2)} < 0.0001$, and **c** AR (n = 3 independent replicates) in H_2O_2 treated mMSCs, $p_{(H2O2)} < 0.0001$, $p_{(Ru\text{-}Cu/EDHJ\text{-}H2O2)} < 0.0001$. Data are presented as means ± SD., $^{***}p < 0.001$, $^{\#\#\#}p < 0.001$; one-way ANOVA with multiple comparisons test. Source data are provided as a Source Data file.

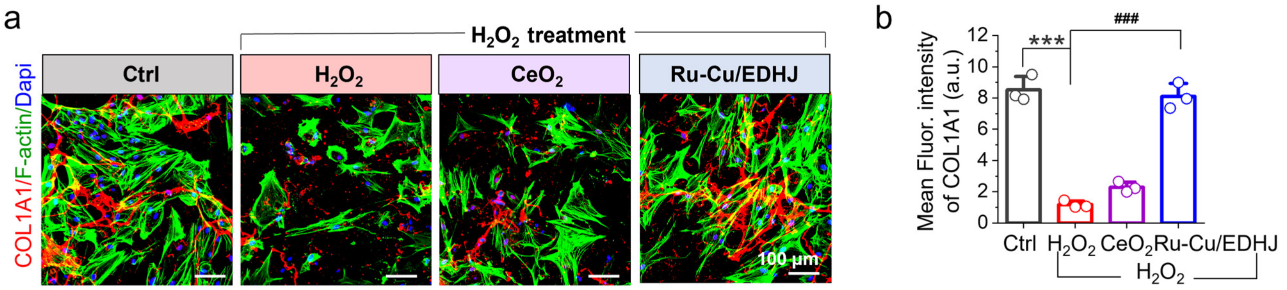

Supplementary Fig. 39. a Fluorescence images of COL1A1. Scale bar: 100 μm. **b** Quantitative results of fluorescence intensity from COL1A1 (n = 3 independent replicates), $p_{(H2O2)} < 0.0001$, $p_{(Ru\text{-}Cu/EDHJ\text{-}H2O2)} < 0.0001$. Data are presented as means ± SD., $^{***}p < 0.001$, $^{\#\#\#}p < 0.001$, one-way ANOVA with multiple comparisons test. Source data are provided as a Source Data file.

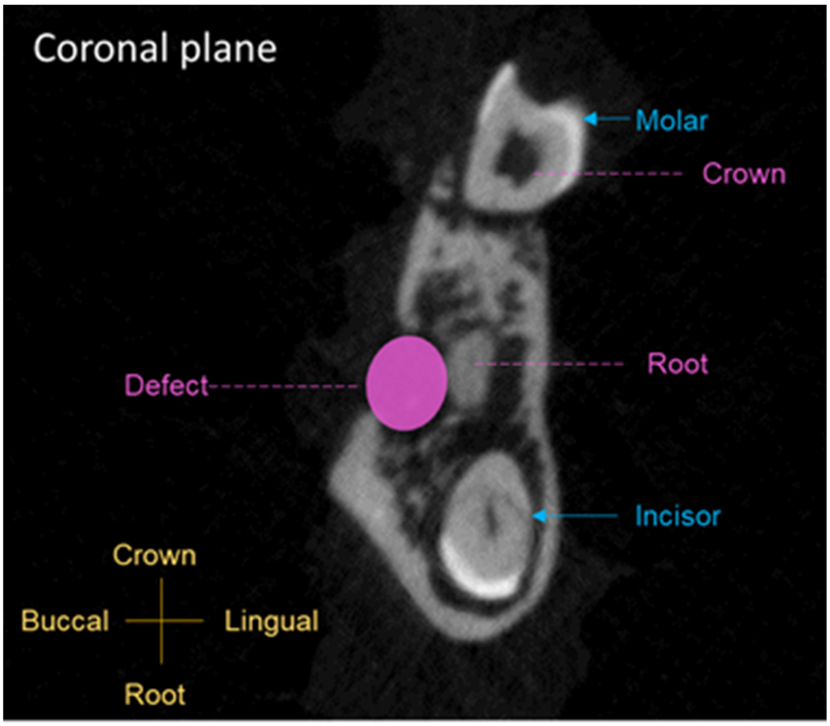

Supplementary Fig. 40. The coronal plane of the mouse mandible by a micro-CT image to show the jaw defect model.

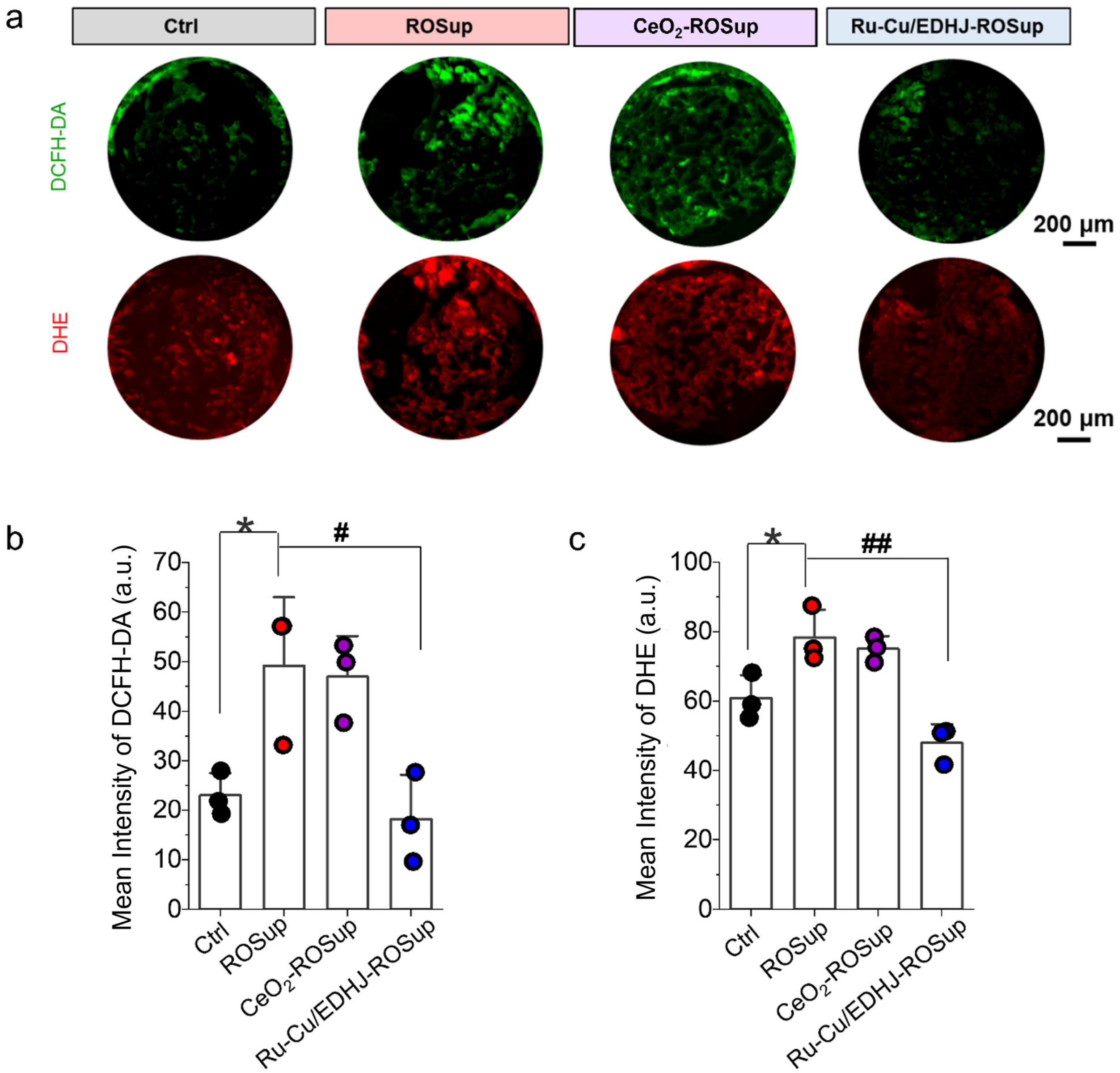

Supplementary Fig. 41. a Fluorescence staining images of 2,7-dichlorodihydrofluorescein diacetate (DCFH-DA) and dihydroethidium (DHE) at week 1 after operation. ROSup means ROS raised up after LPS stimulation. **b** Quantitative results of fluorescence intensity of DCFH-DA (n = 3 independent replicates), $p_{(ROSup)}$ = 0.0396, $p_{(Ru\text{-}Cu/EDHJ\text{-}ROSup)}$ = 0.0166. **c** Quantitative results of fluorescence intensity of DHE (n = 3 independent replicates), $p_{(ROSup)}$ = 0.0340, $p_{(Ru\text{-}Cu/EDHJ\text{-}ROSup)}$ = 0.0014. Ctrl (mandible defects with PBS treatment). Data are presented as means ± SD., $^{*}p < 0.05$, $^{\#}p < 0.05$, $^{\#\#}p < 0.01$; one-way ANOVA with multiple comparisons test. Scale bars: 200 μm. Source data are provided as a Source Data file.

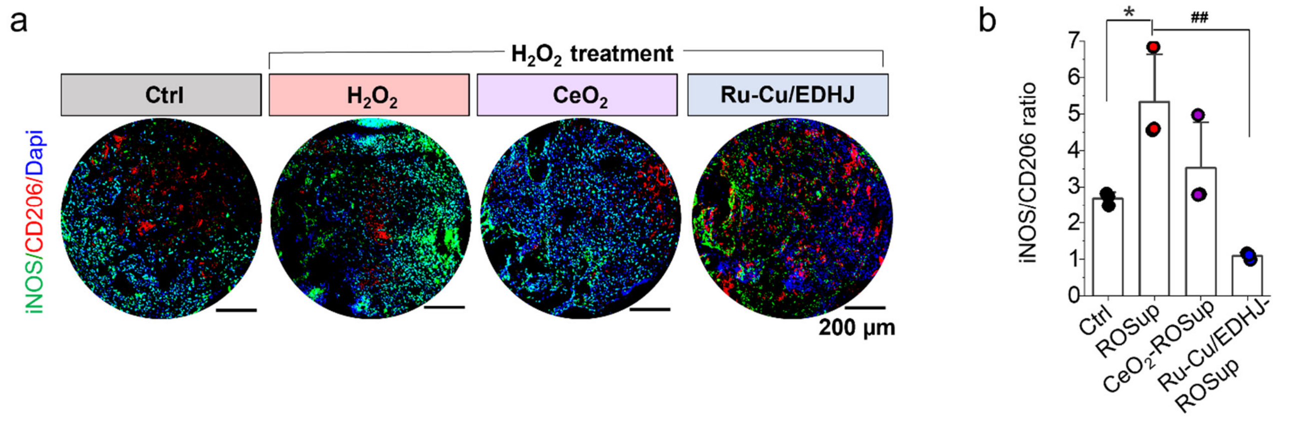

Supplementary Fig. 42. a Double immunofluorescence staining of tissue sections from mandibular defect area for iNOS and CD206 markers, and **b** corresponding quantitative analysis of ratio of iNOS/CD206 (n = 3 independent replicates), $p_{(ROSup)}$ = 0.0304, $p_{(Ru\text{-}Cu/EDHJ\text{-}ROSup)}$ = 0.0021. Ctrl (mandible defects with PBS treatment). Data are presented as means ± SD., $^{*}p < 0.05$, $^{\#\#}p < 0.01$, one-way ANOVA with multiple comparisons test. Scale bars: 200 μm. Source data are provided as a Source Data file.

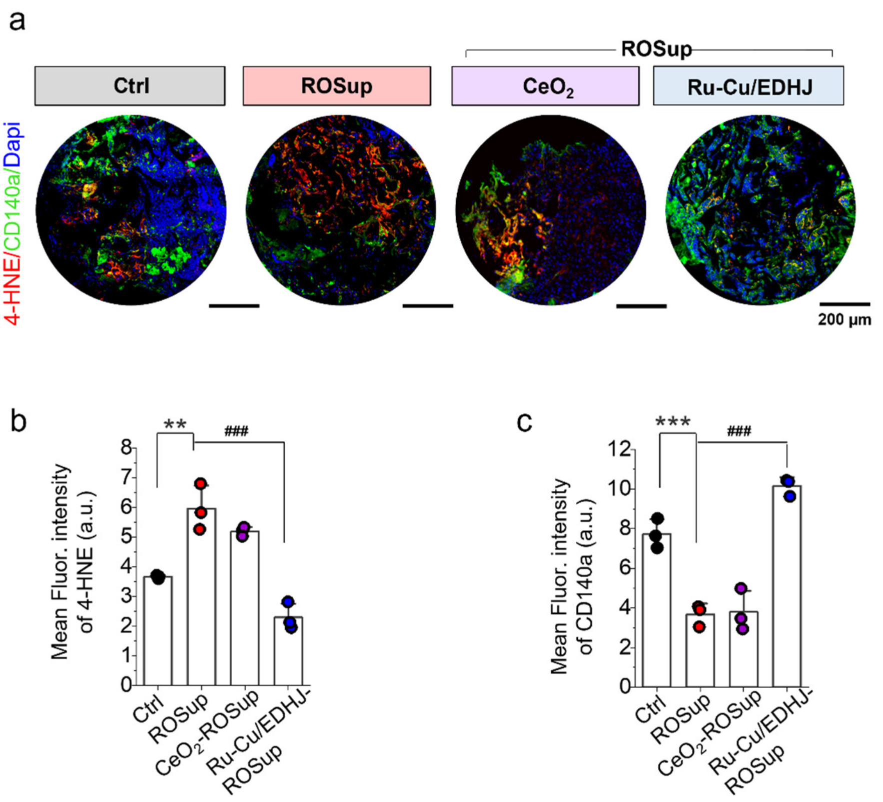

Supplementary Fig. 43. a Fluorescence staining images of 4-hydroxynonenal (4-HNE) and CD140a (platelet-derived growth factor receptor alpha), at week 1 after operation. **b** Quantitative results of

fluorescence intensity of 4-HNE (n = 3 independent replicates), $p_{(ROSup)} = 0.0012$, $p_{(Ru\text{-}Cu/EDHJ\text{-}ROSup)} < 0.0001$. **c** Quantitative results of fluorescence intensity of CD140a (n = 3 independent replicates), $p_{(ROSup)} = 0.0007$, $p_{(Ru\text{-}Cu/EDHJ\text{-}ROSup)} < 0.0001$. Ctrl (mandible defects with PBS treatment). Data are presented as means ± SD., $^{**}p < 0.01$, $^{***}p < 0.001$, $^{\#\#\#}p < 0.001$; one-way ANOVA with multiple comparisons test. Scale bars: 200 μm. Source data are provided as a Source Data file.

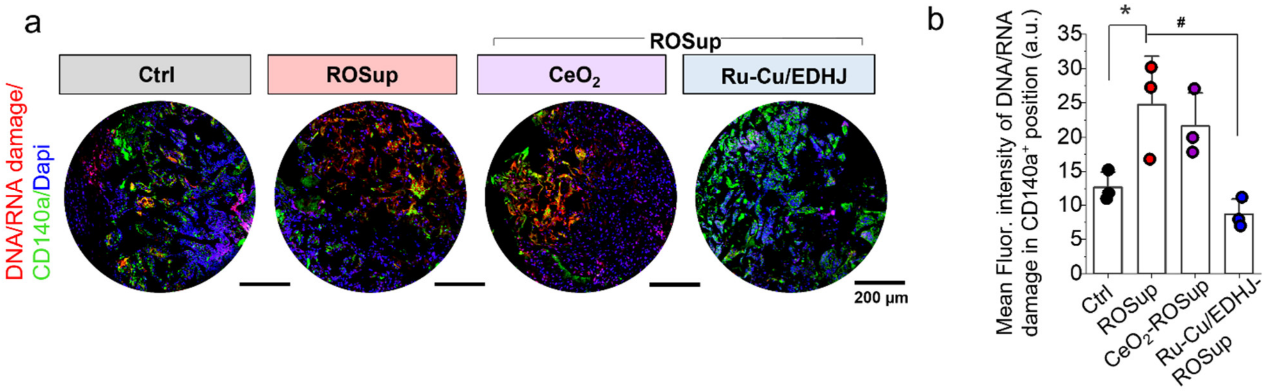

Supplementary Fig. 44. a Fluorescence staining images of DNA/RNA damage and CD140a (platelet-derived growth factor receptor alpha), at week 1 after operation. **b** Quantitative results of mean fluorescence intensity of DNA/RNA damage in $CD140a^+$ position (n = 3 independent replicates), $p_{(ROSup)} = 0.0469$, $p_{(Ru\text{-}Cu/EDHJ\text{-}ROSup)} = 0.0110$. Ctrl (mandible defects with PBS treatment). Data are presented as means ± SD., $^{*}p < 0.05$, $^{\#}p < 0.05$, one-way ANOVA with multiple comparisons test. Scale bars: 200 μm. Source data are provided as a Source Data file.

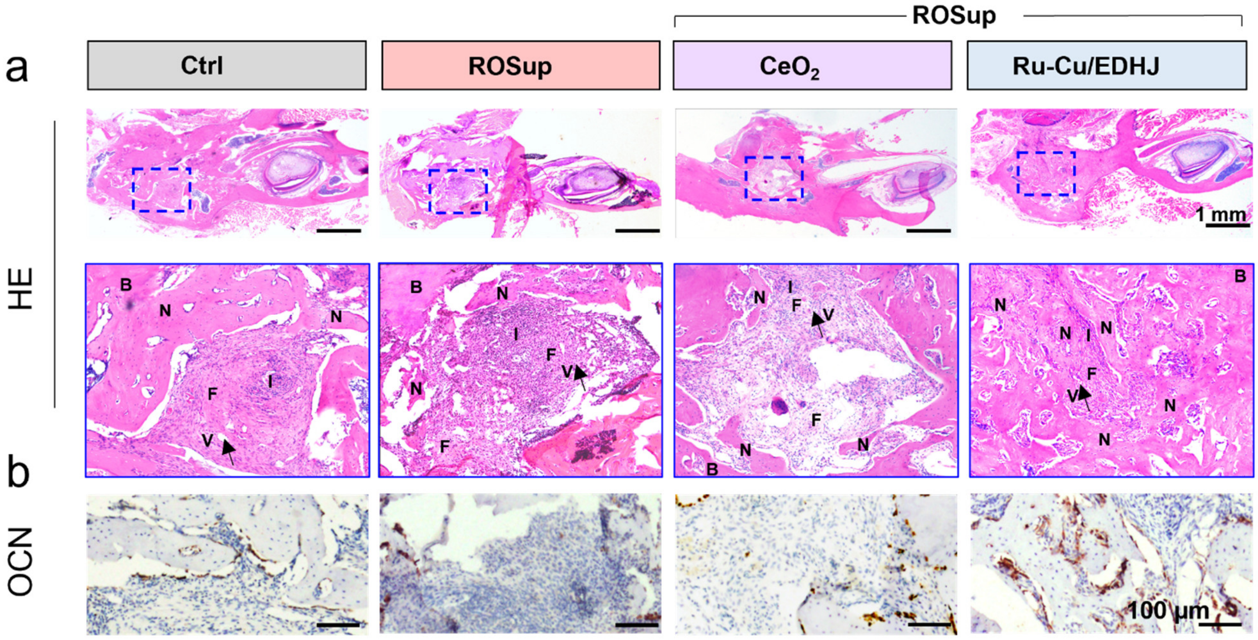

Supplementary Fig. 45. a Hematoxylin and Eosin (H&E) staining of regenerated bones induced by different scaffolds at week 8 after operation. Ctrl (mandible defects with PBS treatment). (Row 1: Overall observation of the mandible defect repair. Row 2: Magnified view of the center and boundary site of the defects). (N: new bone tissue. V: new blood vessels (black arrow). F: fibrous tissue. B: old bone. I: inflammatory infiltration). **b** Representative immunohistochemistry images of OCN. OCN (brown) and hematoxylin (blue). n = 3 independent replicates.

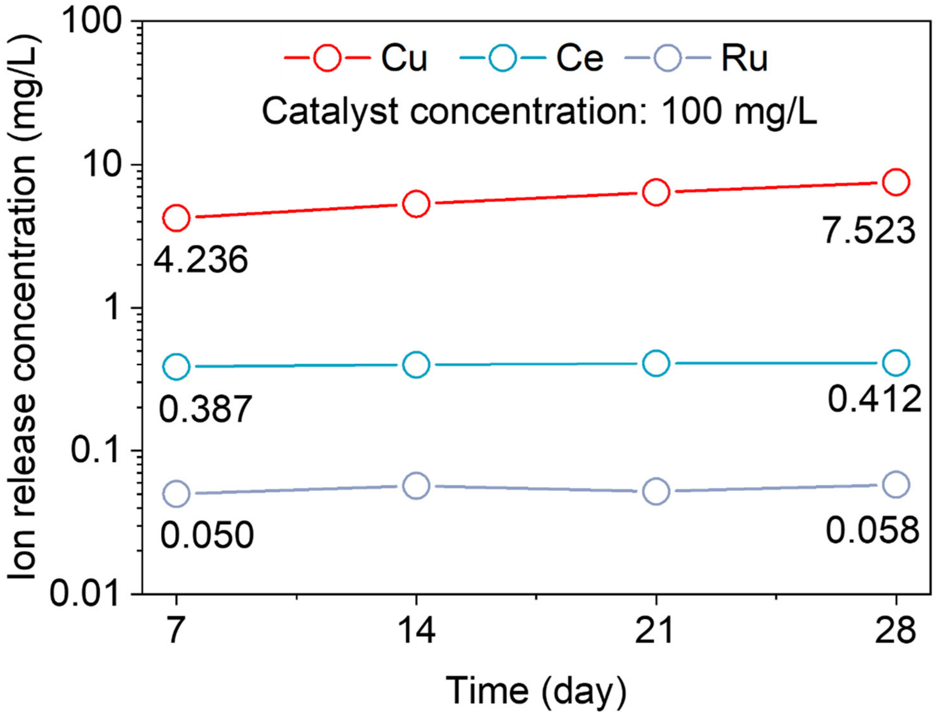

Supplementary Fig. 46. Inductively coupled plasma mass spectrometry (ICP-MS) was used to measure the concentration of ions released at different times. 1 mg of the Ru-Cu/EDHJ was immersed in 10 mL PBS to explore the degradation performance of the material over time *in vitro*. Source data are provided as a Source Data file.

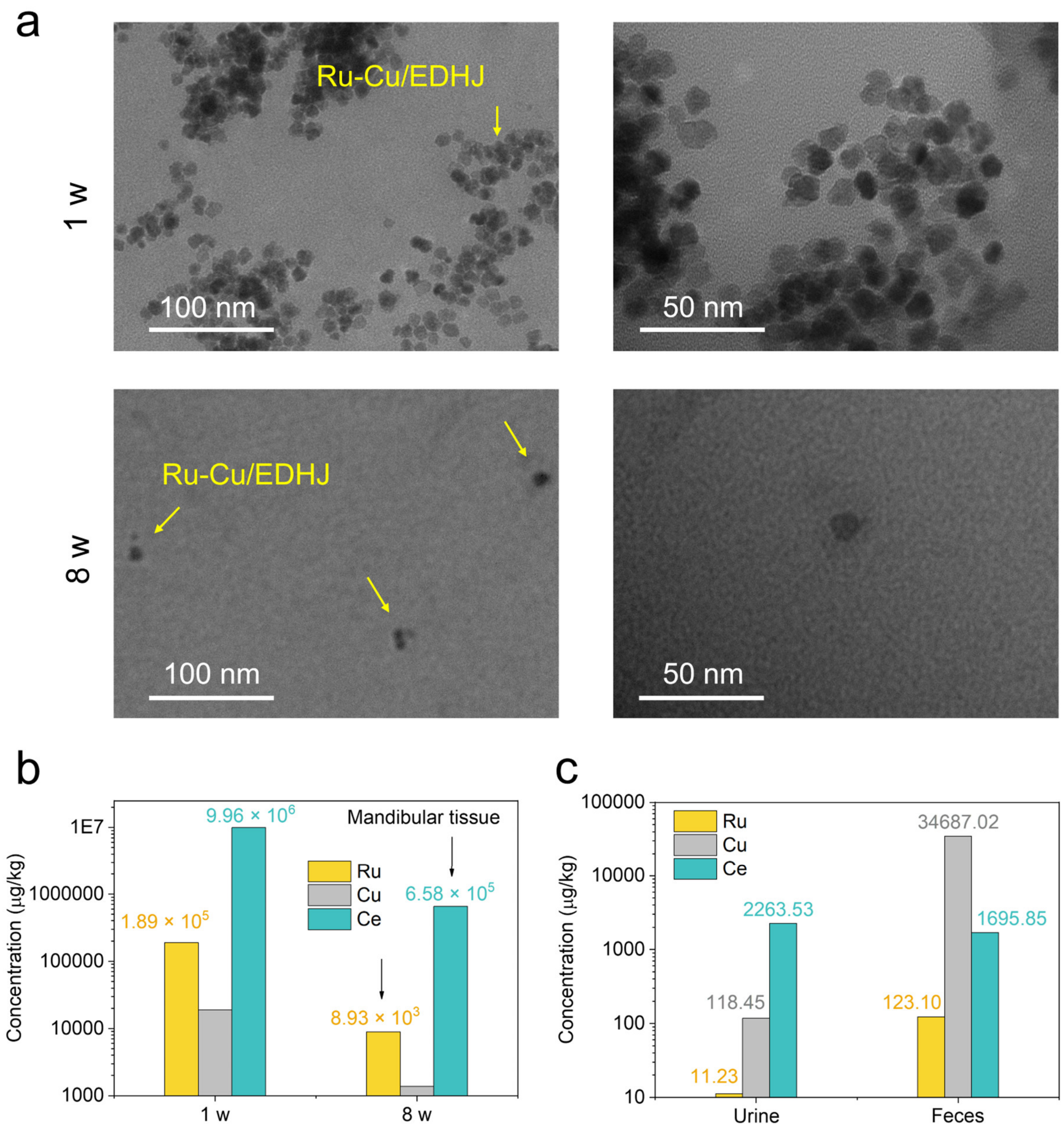

Supplementary Fig. 47. a The TEM images showcase the Ru-Cu/EDHJ materials obtained from the mandibular tissues captured at various time points after the implantation for 1 week and 8 weeks. Mandibular tissue samples were first fixed in osmium tetroxide, then dehydrated in acetone, followed by embedding in epoxy resin, and finally sectioned into ultra-thin slices for TEM imaging. **b** The ICP-MS test measured different metal ion concentrations in mandibular tissue after the implantation of the Ru-Cu/EDHJ for 1 week and 8 weeks. **c** The ICP-MS test measured different metal ion concentrations in mouse urine and feces after the implantation of the Ru-Cu/EDHJ for 4 weeks. Source data are provided as a Source Data file.

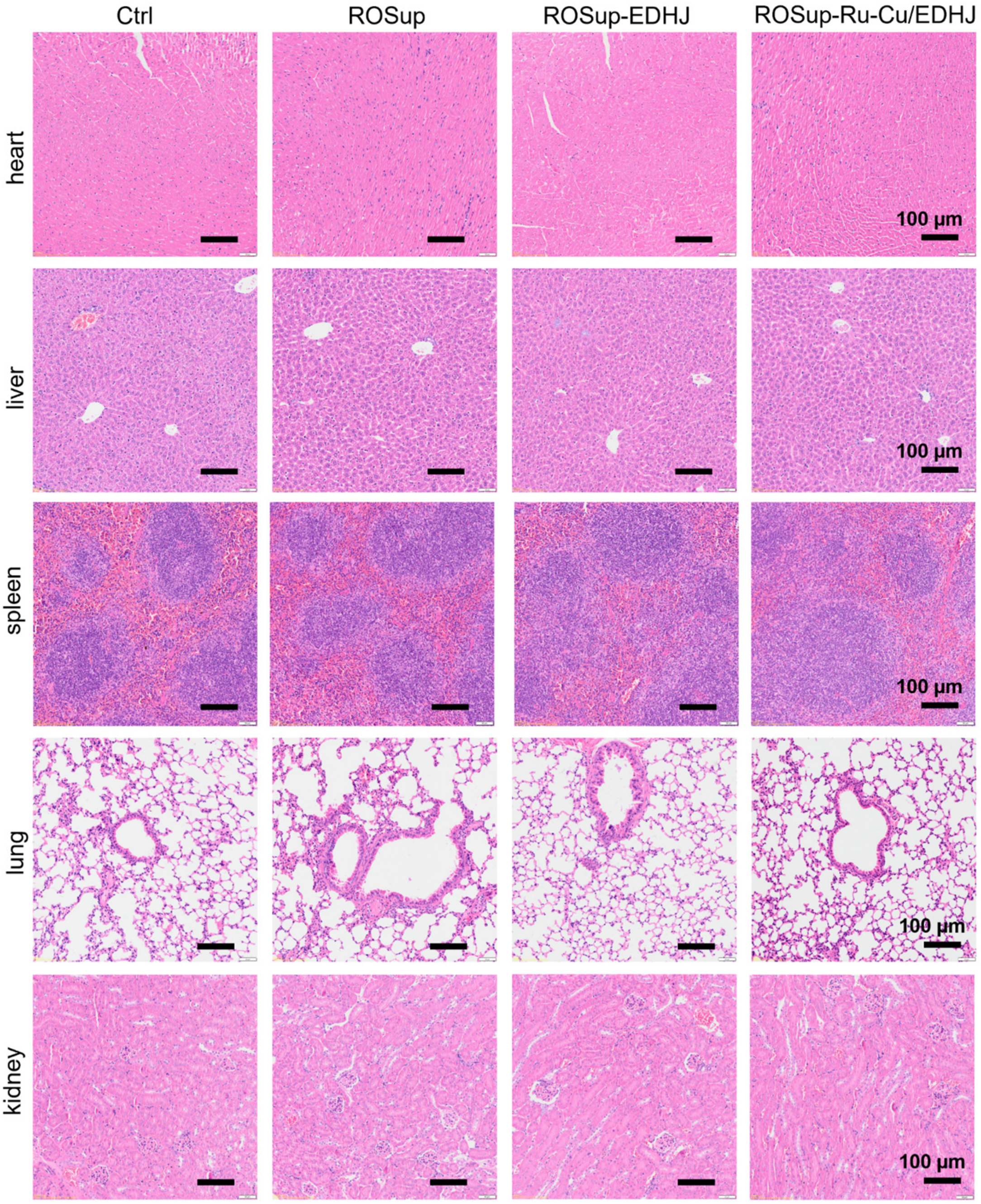

Supplementary Fig. 48. Paraffin-embedded heart, liver, spleen, lung, and kidney of mice on 14 days post-operation were sectioned and stained by H&E (n = 3 independent replicates). Scale bars: 100 μm.

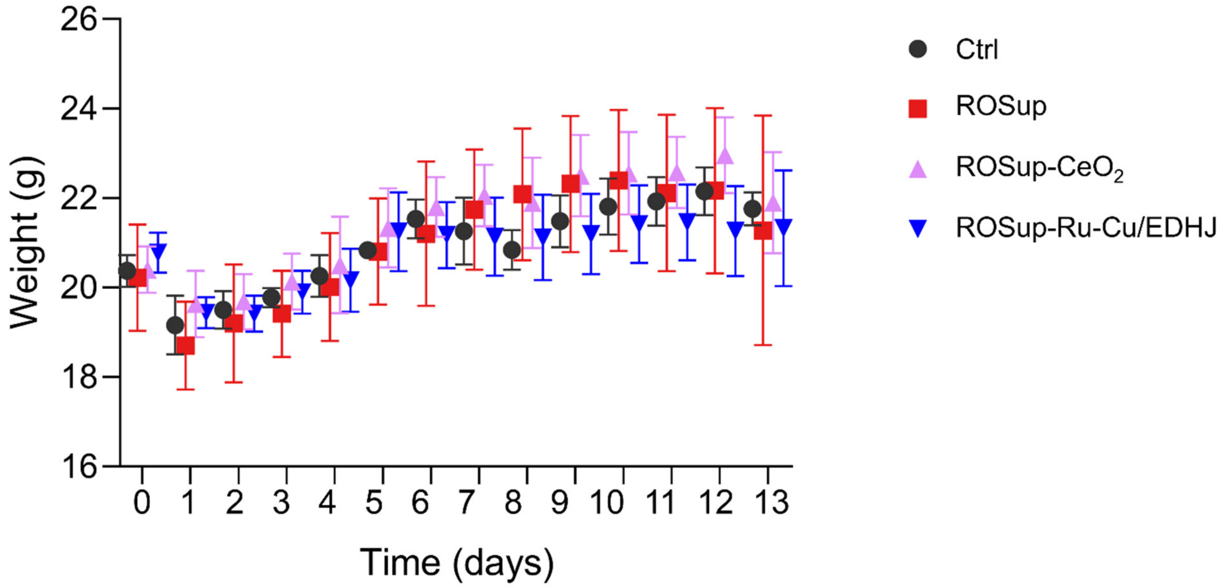

Supplementary Fig. 49. Quantification analysis of the weight of mice in different groups after operation for 14 days continuously (n = 3 independent samples). Source data are provided as a Source Data file.

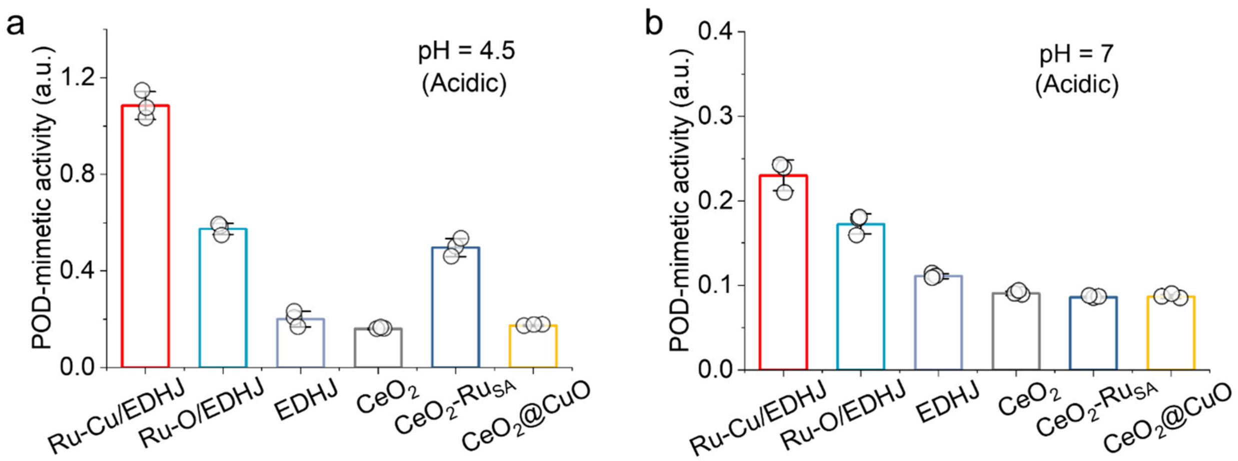

Supplementary Fig. 50. Peroxidase (POD)-mimetic activities of different catalysts at **a** pH 4.5 and **b** pH 7 (n = 3 independent replicates). All error bars represent mean ± SD. Source data are provided as a Source Data file.

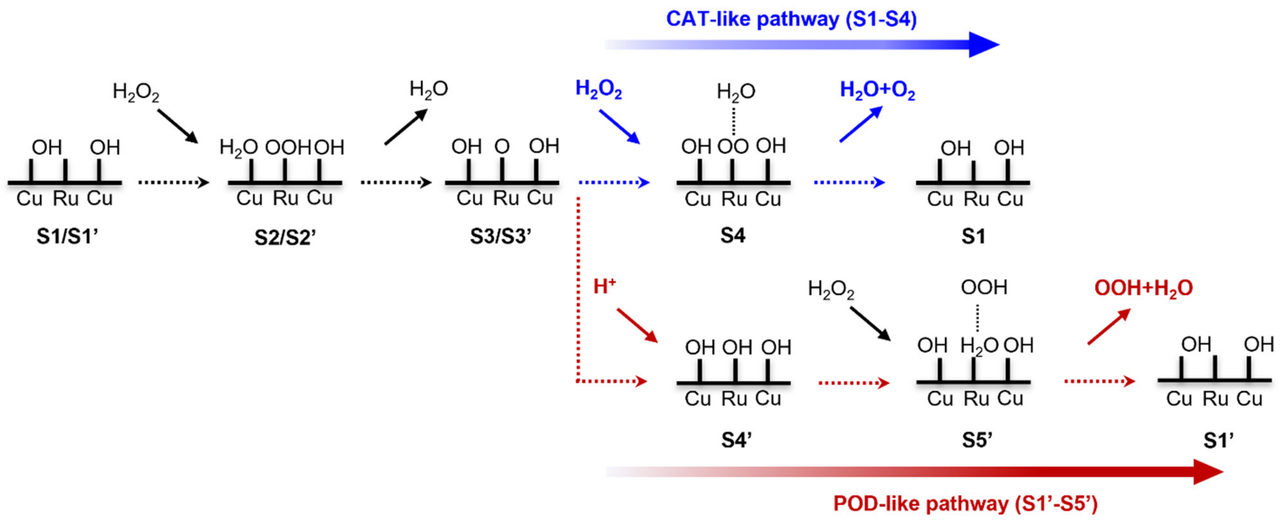

Supplementary Fig. 51. CAT-like and POD-like pathways of Ru-Cu/EDHJ. The Ru-Cu/EDHJ catalyst has demonstrated the ability to spontaneously catalyze H_2O_2, generating a significant quantity of *OH intermediates (with Gibbs free energy of -3.658 eV) that promote the formation of OH-enriched surfaces. Consequently, we employed *OH + *OH as the reaction initial state (**S1/S1'**) in this part.

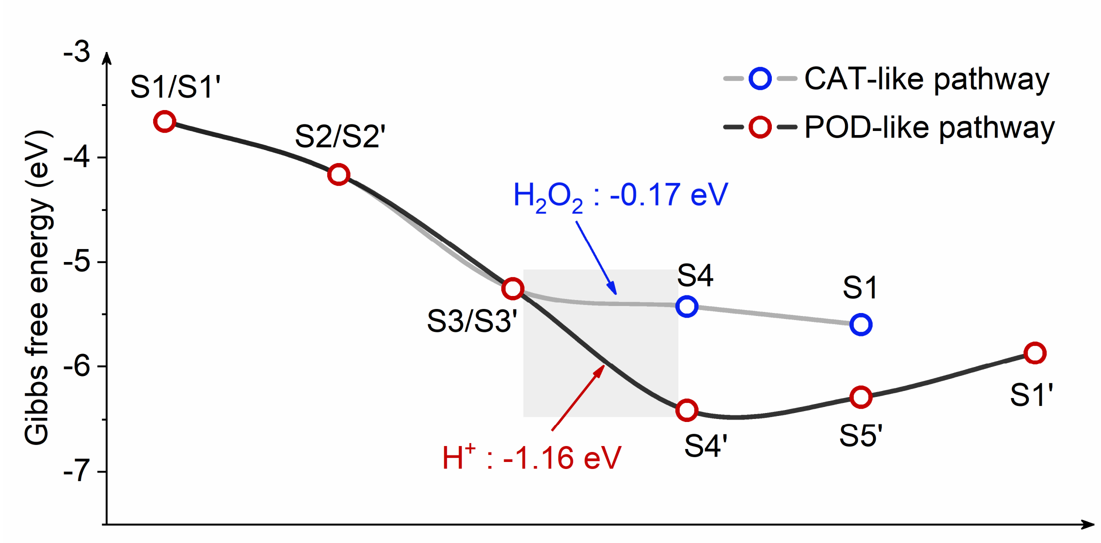

Supplementary Fig. 52. Gibbs free energy diagram of corresponding CAT-like and POD-like pathways for Ru-Cu/EDHJ. Source data are provided as a Source Data file.

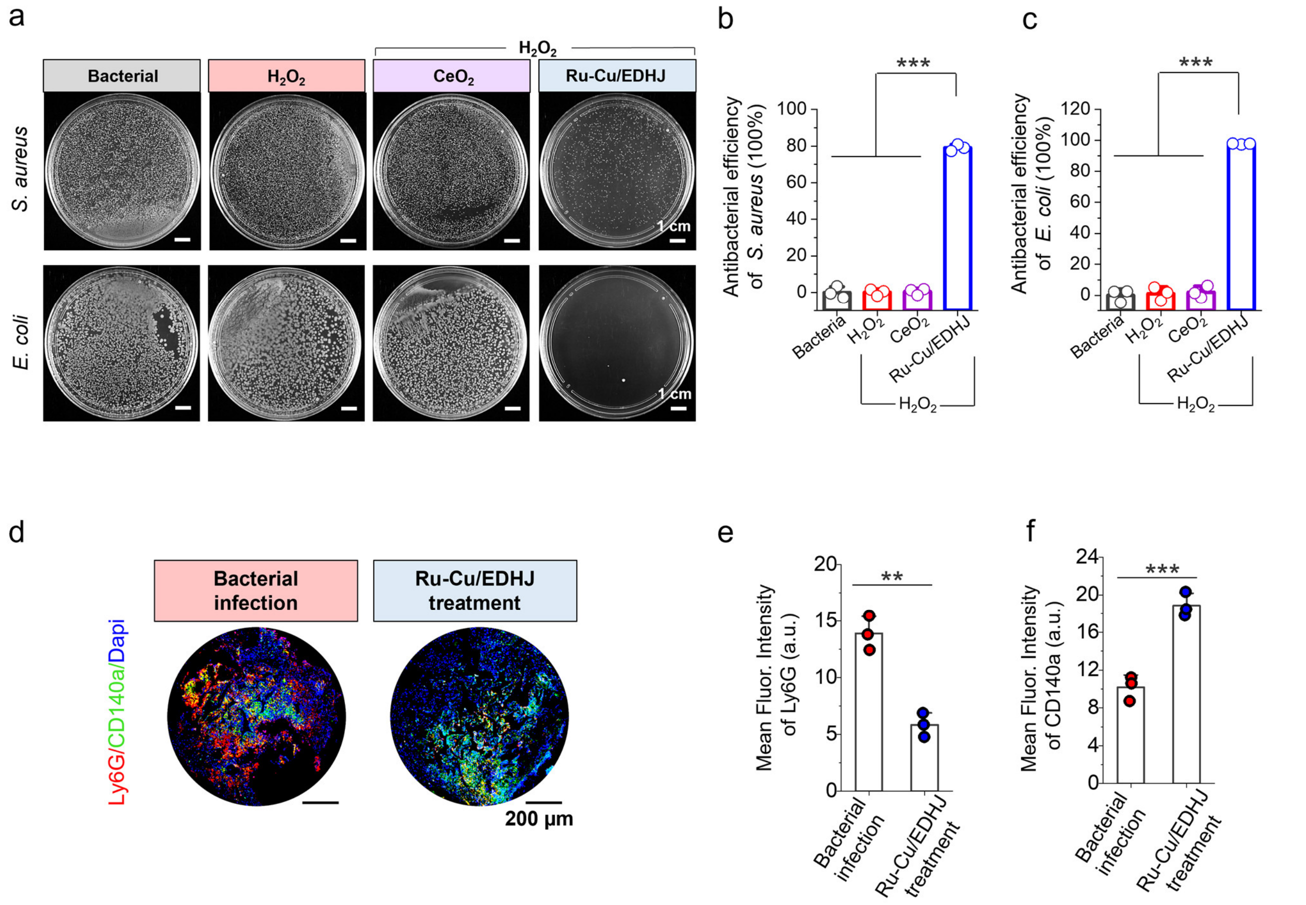

Supplementary Fig. 53. **a** Bacterial colonies formed by *S. aureus* and *E. coli* after incubating with PBS, H_2O_2, $CeO_2 + H_2O_2$, or Ru-Cu/EDHJ + H_2O_2. **b** The corresponding antibacterial efficiency of *S. aureus* (n = 3 independent replicates), $p_{(Bacteria)} < 0.0001$, $p_{(H2O2)} < 0.0001$, $p_{(CeO2\text{-}H2O2)} < 0.0001$. Data are presented as means ± SD., $^{***}p < 0.001$, one-way ANOVA with multiple comparisons test. **c** The corresponding antibacterial efficiency of *E. coli* (n = 3 independent replicates), $p_{(Bacteria)} < 0.0001$, $p_{(H2O2)} < 0.0001$, $p_{(CeO2\text{-}H2O2)} < 0.0001$. Data are presented as means ± SD., $^{***}p < 0.001$, one-way ANOVA with multiple comparisons test. **d** Fluorescence staining images of Ly6G and CD140a at week 1 after operation. **e** Quantitative results of fluorescence intensity of Ly6G (n = 3 independent replicates), $p_{(Ru\text{-}Cu/EDHJ)} = 0.0016$. Data are presented as means ± SD., $^{**}p < 0.01$, p values are assessed by unpaired Student's two-sided t-tests. **f** Quantitative results of fluorescence intensity of CD140a (n = 3 independent replicates), $p_{(Ru\text{-}Cu/EDHJ)} = 0.0010$. Data are presented as means ± SD., $^{***}p < 0.001$, p values are assessed by unpaired Student's two-sided t-tests. All antibacterial experiments are performed in acidic conditions (PBS: pH 5.6). Source data are provided as a Source Data file.

Supplementary Tables

Supplementary Table 1. The elemental contents in the biocatalysts that are determined by XPS measurements.

XPS	Atomic(%)				Weight(%)			
	Cu	Ce	O	Ru	Cu	Ce	O	Ru
CeO_2	-	24.27	75.73	-	-	73.73	26.27	-
CeO_2-Ru_{SA}	-	21.02	75.64	3.34	-	65.55	26.94	7.51
CeO_2@CuO	4.03	6.64	89.33	-	9.79	35.57	54.64	-
EDHJ	10.32	17.39	72.29	-	15.43	57.35	27.22	-
Ru-O/EDHJ	3.47	3.27	90.86	2.40	9.28	19.29	61.21	10.21
Ru-Cu/EDHJ	8.57	11.99	76.67	2.78	14.59	45.01	32.87	7.53

Supplementary Table 2. EXAFS fitting parameters at the Ru *K*-edge for various samples ($S_0^2 = 0.96$). ^aN: coordination numbers; ^bR: bond distance; $^c\sigma^2$: Debye-Waller factors; $^d\Delta E_0$: the inner potential correction. *R* factor: goodness of fit. S_0^2 was set to 0.96, according to the experimental EXAFS fit of Ru foil by fixing CN as the known crystallographic value.

Sample	Shell	N^a	$R(Å)^b$	$\sigma^2(Å^2)^c$	$\Delta E_0(eV)^d$	*R* factor
Ru-Cu/EDHJ	Ru-O	1.98	2.02	0.0069	1.11	0.0168
	Ru-Cu	1.00	2.44	0.0079		
	Ru-Cu	1.00	2.65	0.0020		
Ru-O/EDHJ	Ru-O	1.97	1.97	0.0017	0.12	0.0061
	Ru-O	2.06	2.06	0.0031		

Supplementary Table 3. Comparison of the kinetics based on Ru active sites on CeO_2-Ru_{SA}, Ru-O/EDHJ, and Ru-Cu/EDHJ.

Biocatalysts	E_0 (μM)	V_{max} (μM s^{-1})	K_m (mM)	TON (s^{-1})	TON/K_m (s^{-1} M^{-1})
Ru-Cu/EDHJ	7.45	60.24	79.37	8.09	101.88
Ru-O/EDHJ	10.11	21.56	294.72	2.13	7.24
CeO_2-Ru_{SA}	7.43	14.60	212.84	1.96	9.23
