# Supplementary material for: Electron-donable heterojunctions with synergetic Ru-Cu pair sites for biocatalytic microenvironment modulations in inflammatory mandible defects
Source: Nat Commun. 2024 Nov 6;15:9592. doi: 10.1038/s41467-024-53824-y (PMC11541594; doi:10.1038/s41467-024-53824-y)
Supplement: Supplementary file 3 — Description of Additional Supplementary File [file 41467_2024_53824_MOESM3_ESM.pdf]

Description of Additional supplementary files

Supplementary Data 1: Atomic coordinates of the optimized computational models of Cu2O-RuSA active component in Ru-Cu/EDHJ.

Supplementary Data 2: Atomic coordinates of the optimized computational models of CuO-RuSA active component in Ru-O/EDHJ.

Supplementary Data 3: Atomic coordinates of the optimized computational models of CeO2-RuSA.

Supplementary Data 4: Comparison of Vmax and TON with recently reported state-of-the-art ROS-scavenging biocatalysts.
