# Supplementary material for: Electron-donable heterojunctions with synergetic Ru-Cu pair sites for biocatalytic microenvironment modulations in inflammatory mandible defects
Source: Nat Commun. 2024 Nov 6;15:9592. doi: 10.1038/s41467-024-53824-y (PMC11541594; doi:10.1038/s41467-024-53824-y)
Supplement: Supplementary file 8 — Reporting Summary [file 41467_2024_53824_MOESM8_ESM.pdf]

nature portfolio

Corresponding author(s): Chong Cheng
Last updated by author(s): Oct 9, 2024

Reporting Summary

Nature Portfolio wishes to improve the reproducibility of the work that we publish. This form provides structure for consistency and transparency in reporting. For further information on Nature Portfolio policies, see our Editorial Policies and the Editorial Policy Checklist.

Statistics

For all statistical analyses, confirm that the following items are present in the figure legend, table legend, main text, or Methods section.

n/a	Confirmed	
☐	☒	The exact sample size (*n*) for each experimental group/condition, given as a discrete number and unit of measurement
☐	☒	A statement on whether measurements were taken from distinct samples or whether the same sample was measured repeatedly
☐	☒	The statistical test(s) used AND whether they are one- or two-sided<br>*Only common tests should be described solely by name; describe more complex techniques in the Methods section.*
☒	☐	A description of all covariates tested
☒	☐	A description of any assumptions or corrections, such as tests of normality and adjustment for multiple comparisons
☐	☒	A full description of the statistical parameters including central tendency (e.g. means) or other basic estimates (e.g. regression coefficient) AND variation (e.g. standard deviation) or associated estimates of uncertainty (e.g. confidence intervals)
☐	☒	For null hypothesis testing, the test statistic (e.g. *F*, *t*, *r*) with confidence intervals, effect sizes, degrees of freedom and *P* value noted<br>*Give P values as exact values whenever suitable.*
☒	☐	For Bayesian analysis, information on the choice of priors and Markov chain Monte Carlo settings
☒	☐	For hierarchical and complex designs, identification of the appropriate level for tests and full reporting of outcomes
☒	☐	Estimates of effect sizes (e.g. Cohen's *d*, Pearson's *r*), indicating how they were calculated

Our web collection on statistics for biologists contains articles on many of the points above.

Software and code

Policy information about availability of computer code

Data collection	The structure analysis of materials was measured by the X-ray diffraction (DX-2700BH, HaoYuan Instrument, China), X-ray photoelectron spectroscopy (Thermo Scientific), and X-ray absorption spectroscopy (Taiwan Photon Source, National Synchrotron Radiation Research Center). The surface morphology and dimensions analysis of materials were conducted with a Hitachi Regulus8220 field emission scanning electron microscopy and a FEI Talos F200X transmission electron microscopy. Aberration-corrected high-angle annular dark-field scanning transmission electron microscopy and energy dispersive spectroscopy mapping were performed via FEI Titan Cubed Themis G2 300 and Titan Themis 60-300 operated at 200 kV. For bone defects, transmission electron microscopy was conducted using a Talos F200S microscope. The absorbance was detected by a multifunctional enzyme labeling instrument (ReadMax1900). Fluorescence images were collected via a confocal laser scanning microscope (FV3000 and Nikon, Olympus, Japan). Live/dead staining was counted using a Celigo Image Cytometer (Nexcelom Bioscience LCC., USA). Flow-cytometry data were collected via a flow cytometer (AttuneTM NxT, Invitrogen, USA). RNA concentration and purity were measured using NanoDrop 2000 (Thermo Fisher Scientific, Wilmington, DE, USA). RNA integrity was assessed using the RNA Nano 6000 Assay Kit of the Agilent Bioanalyzer 2100 system (Agilent Technologies, CA, USA). Alkaline Phosphatase and Alizarin Red staining images were taken with a stereomicroscope (SZX16, Olympus, Japan). H&E staining and reactive oxygen species determination in vivo were scanned by a full slide scanner (VS200, Olympus, Japan). Micro-CT images were collected using µ-CT Scanner (µ-CT50, Scanco, Bassersdorf, Zurich, Switzerland). The libraries were sequenced on an Illumina NovaSeq 6000 platform.
Data analysis	Data were analyzed using GraphPad Prism 8.0, Origin 2022, VASP 5.4.1, MDI Jade 6, Adobe Illustrator 27.0.1, Avantage 5.967, Athena software 0.9.26, Artemis software 0.9.26, and Digital Micrograph 3.7.4. The software Image J 1.8.0 and Image-Pro Plus 6.0 were used for in vitro and in vivo imaging analysis. Flow-cytometry analysis was performed in FlowJo v10.8.1 and in FlowJo v10.8.1 or in the software of AttuneTM NxT. Bioinformatic analysis was performed using the free online platform BMKCloud (www.biocloud.net).

For manuscripts utilizing custom algorithms or software that are central to the research but not yet described in published literature, software must be made available to editors and reviewers. We strongly encourage code deposition in a community repository (e.g. GitHub). See the Nature Portfolio guidelines for submitting code & software for further information.

Data

Policy information about availability of data

All manuscripts must include a data availability statement. This statement should provide the following information, where applicable:

- Accession codes, unique identifiers, or web links for publicly available datasets
- A description of any restrictions on data availability
- For clinical datasets or third party data, please ensure that the statement adheres to our policy

The main data supporting the results of this study are available within the paper and its Supplementary Information. Any other raw data or noncommercial material used in this study are available from the corresponding author. Raw RNA sequencing data generated in this study have been deposited in the NCBI SRA database under accession number GSE253527 [https://www.ncbi.nlm.nih.gov/geo/query/acc.cgi?acc=GSE253527]. Source data are provided with this paper.

Research involving human participants, their data, or biological material

Policy information about studies with human participants or human data. See also policy information about sex, gender (identity/presentation), and sexual orientation and race, ethnicity and racism.

Reporting on sex and gender	N/A
Reporting on race, ethnicity, or other socially relevant groupings	N/A
Population characteristics	N/A
Recruitment	N/A
Ethics oversight	N/A

Note that full information on the approval of the study protocol must also be provided in the manuscript.

Field-specific reporting

Please select the one below that is the best fit for your research. If you are not sure, read the appropriate sections before making your selection.

☒ Life sciences ☐ Behavioural & social sciences ☐ Ecological, evolutionary & environmental sciences

For a reference copy of the document with all sections, see nature.com/documents/nr-reporting-summary-flat.pdf

Life sciences study design

All studies must disclose on these points even when the disclosure is negative.

Sample size	All biologically based assays were performed with the usual and sufficient sample size setting according to earlier paper (Nat. Commun. 2021, 12(1), 6143). These sample sizes were sufficient for a statistical analysis. All experiments reported here have n number and repetitions reported.
Data exclusions	No data was excluded from the analysis.
Replication	Results shown in the manuscript are representative of at least three independent experiments. All our attempts at replication were successful with similar results.
Randomization	Our samples/organisms were allocated randomly.
Blinding	In all experiments, investigators were blinded to group allocation during data collection and processing.

Reporting for specific materials, systems and methods

We require information from authors about some types of materials, experimental systems and methods used in many studies. Here, indicate whether each material, system or method listed is relevant to your study. If you are not sure if a list item applies to your research, read the appropriate section before selecting a response.

Materials & experimental systems

n/a	Involved in the study
☐	☒ Antibodies
☒	☐ Eukaryotic cell lines
☒	☐ Palaeontology and archaeology
☐	☒ Animals and other organisms
☒	☐ Clinical data
☒	☐ Dual use research of concern
☒	☐ Plants

Methods

n/a	Involved in the study
☒	☐ ChIP-seq
☐	☒ Flow cytometry
☒	☐ MRI-based neuroimaging

Antibodies

Antibodies used

Primary antibodies and corresponding concentrations used in this study were 4-HNE (MA5-27570, Life Technologies, USA,1:100 dilution), paxillin (ab32084, Abcam, USA, 1:200 dilution), γH2A.X (phospho S139) (ab81299, Abcam, USA, 1:200 dilution), DNA/RNA Damage (ab62623, Abcam, USA, 1:200 dilution), and COL1A1 (R26615, ZEN-BIOSCIENCE, China, 1:50 dilution), TNFα (346654, ZEN-BIOSCIENCE, China, 1:50 dilution), BMP2/4 (sc-137087, Santa Cruz Biotechnology, USA, 1:50 dilution), CD140a (ab203491, Abcam, USA, 1:200 dilution), iNOS (GB11119, Servicebio, China, 1:50), CD206 (sc-58986, Santa Cruz Biotechnology, USA, 1:50 dilution), Ly6g (GB11229, Servicebio, China, 1:50), OCN (GB11233, Servicebio, China, 1:200). The cytoskeleton was stained by FITC-conjugated phalloidin (A12379, Invitrogen, USA, 1:200 dilution). The secondary antibodies were Alexa Fluor 647 goat anti-mouse IgG (ab150115, Abcam, USA, 1:200 dilution), Alexa Fluor 647 donkey anti-rabbit IgG (ab150075, Abcam, USA, 1:200 dilution), Alexa Fluor 488 goat anti-rabbit IgG (ab150077, Abcam, USA, 1:200 dilution), Cy3 goat anti-rabbit IgG (GB21303, Servicebio, China, 1:50), Cy3 goat anti-mouse IgG (GB21301, Servicebio, China, 1:50), and HRP conjugated Goat Anti-Rabbit IgG (GB23303, Servicebio, China, 1:200).

Validation

All antibodies were verified by the supplier and each lot has been quality tested. All validation statements of primary antibodies can be found on the respective antibody website:
4-HNE: https://www.thermofisher.cn/cn/zh/antibody/product/4-Hydroxynonenal-Antibody-clone-12F7-Monoclonal/MA5-27570
paxillin: https://www.abcam.cn/products/primary-antibodies/paxillin-antibody-y113-ab32084.html
γH2A.X: https://www.abcam.cn/products/primary-antibodies/gamma-h2ax-phospho-s139-antibody-ep8542y-ab81299.html
DNA/RNA Damage: https://www.abcam.cn/products/primary-antibodies/dnarna-damage-antibody-15a3-ab62623.html
COL1A1: http://www.zen-bio.cn/prod_view.aspx?TypeId=245&Id=656838&FId=t3:245:3
TNFα: http://www.zen-bio.cn/prod_view.aspx?TypeId=246&Id=681465&FId=t3:246:3
BMP2/4: https://www.scbt.com/zh/p/bmp-2-4-antibody-h-1
CD140a: https://www.abcam.cn/products/primary-antibodies/pdgfr-alpha-antibody-epr22059-270-ab203491.html
iNOS: https://www.servicebio.cn/goodsdetail?id=1394
CD206: https://www.scbt.com/zh/p/cd206-antibody-15-2
Ly6g: https://www.servicebio.cn/goodsdetail?id=1454
OCN: https://www.servicebio.cn/goodsdetail?id=1458
FITC-conjugated phalloidin: https://www.thermofisher.cn/order/catalog/product/A12379?SID=srch-hj-A12379

Animals and other research organisms

Policy information about studies involving animals; ARRIVE guidelines recommended for reporting animal research, and Sex and Gender in Research

Laboratory animals

C57BL/6 mice (male, 6 weeks old) were weighed 15–25g. All mice were maintained under a 12 h light-dark cycle (light on from 8:00 a.m. to 8:00 p.m.) with ad libitum access to food and water. All diets were prepared by Jiangsu-Xietong, Inc. (Nanjing, China), catalogue number: 1010038. The ambient temperature is 20-26 °C and the humidity is 40-70%.

Wild animals

The study did not involve wild animals.

Reporting on sex

Male mice were selected in this study.

Field-collected samples

The study did not involve samples collected from the field.

Ethics oversight

The animal experiments and procedures, including euthanasia, were performed using protocols approved by the Institutional Animal Care and Use Committee at Sichuan University (Number: WCHSIRB-D-2020-361). The study was reviewed and approved by the Laboratory Animal Welfare and Ethics Committee of West China Hospital of Stomatology. All experiments involving animal use were performed in accordance with the ARRIVE guidelines.

Note that full information on the approval of the study protocol must also be provided in the manuscript.

Plants

Seed stocks: N/A

Novel plant genotypes: N/A

Authentication: N/A

Flow Cytometry

Plots

Confirm that:

☒ The axis labels state the marker and fluorochrome used (e.g. CD4-FITC).

☒ The axis scales are clearly visible. Include numbers along axes only for bottom left plot of group (a 'group' is an analysis of identical markers).

☒ All plots are contour plots with outliers or pseudocolor plots.

☒ A numerical value for number of cells or percentage (with statistics) is provided.

Methodology

Sample preparation	After different treatments, hMSCs were digested by trypsin without EDTA, and were washed several times. Then, they were stained by indicated antibodies, followed by flow cytometric analysis.
Instrument	AttuneTM NxT, Invitrogen, USA
Software	Flow-cytometry analysis was performed in FlowJo v10.8.1 or in the software of AttuneTM NxT.
Cell population abundance	At least 5,000 relevant events were acquired for all flow-cytometry analyses .
Gating strategy	In general, cells were first gated on FSC/SSC. Annexin V-FITC and PI gating was performed on the cell population. The cell populations are assigned into four quadrants, including live, early apoptotic, late apoptotic, and necrotic cells.

☒ Tick this box to confirm that a figure exemplifying the gating strategy is provided in the Supplementary Information.
